# Recyclable and bio-based waterborne acetal thermosets and composites

**DOI:** 10.1039/d6gc03576k

**Published:** 2026-08-15

**Authors:** Patrick Schara, Željko Tomović

**Affiliations:** a Polymer Performance Materials Group, Department of Chemical Engineering and Chemistry and Institute for Complex Molecular Systems (ICMS), Eindhoven University of Technology 5600 MB Eindhoven The Netherlands p.j.schara@tue.nl z.tomovic@tue.nl

## Abstract

Fully recyclable waterborne acetal thermosets were synthesized *via* the condensation of sorbitol and glutaraldehyde, utilizing oxalic acid as a green and *in situ* decomposable catalyst. *Via* a simple one-pot polycondensation in water the catalyst-free densely crosslinked networks are formed, combining high mechanical performance (tensile strength >90 MPa), stiffness (Young's modulus 3.3 GPa), and excellent thermal and solvent stability. While the materials remain fully stable under neutral conditions, the acetal linkages enable complete depolymerization under acidic environments, producing a homogeneous solution that can be recondensed into new thermosets with properties indistinguishable from the original, thus achieving true closed-loop recycling. The resins also exhibit outstanding compatibility with glass and carbon fibers, enabling the fabrication of high-performance, recyclable composites from which pristine fibers can be recovered. Together, these results establish waterborne, bio-based acetal chemistry as a scalable and sustainable platform for high-performance thermosets and fiber composites, offering a practical and circular route toward next-generation materials design.

## Abstract 2

Waterborne bio-based acetal thermosets are synthesized from sorbitol and glutaraldehyde using a decomposable catalyst. They exhibit high performance, undergo closed-loop chemical recycling and enable recyclable glass- and carbon-fiber composites.

## Graphical abstract

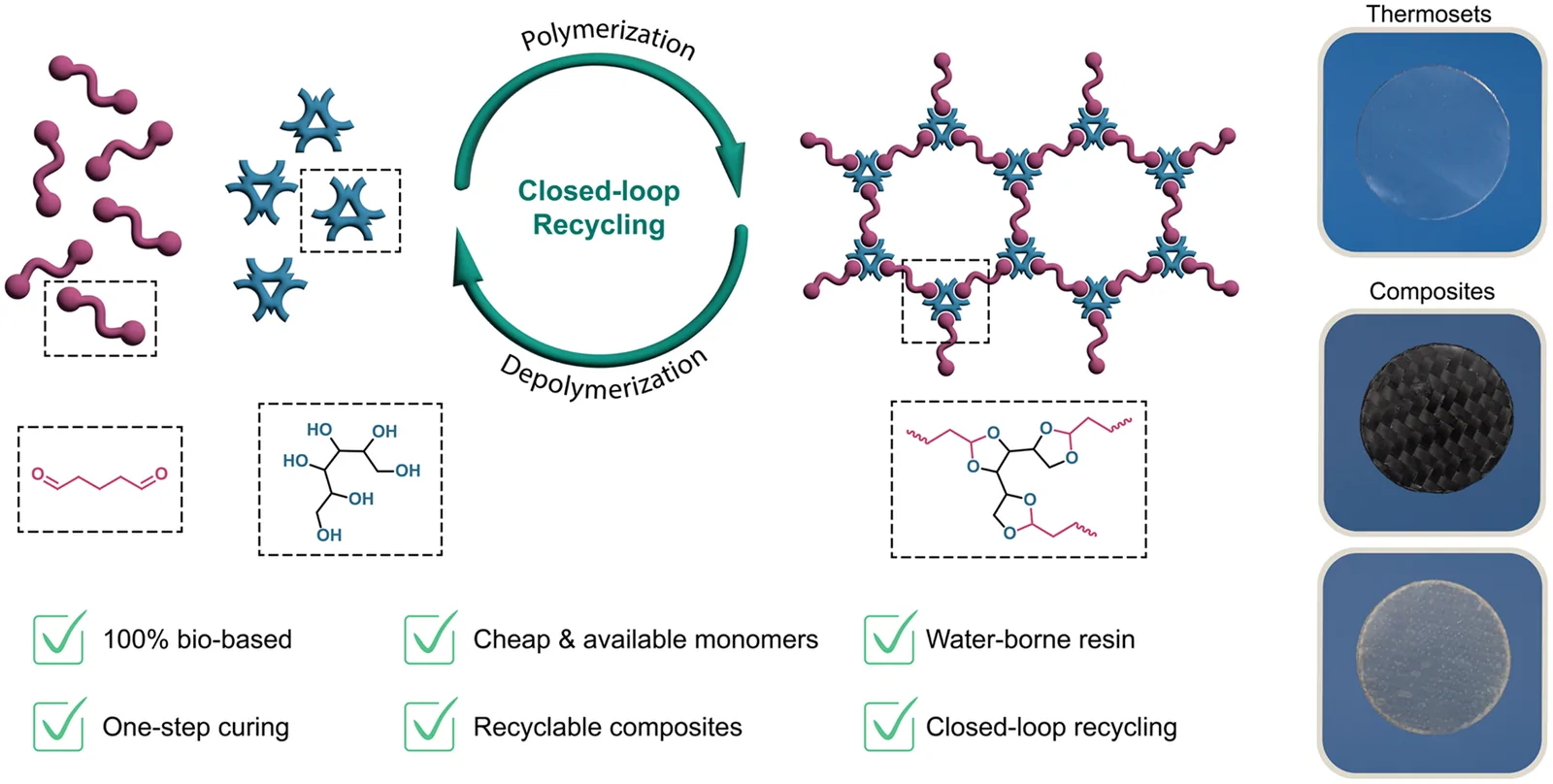

**Green foundation**

1. This work advances green chemistry by introducing a fully bio-based, waterborne thermosetting resin derived from sorbitol and glutaraldehyde that combines high-performance mechanical properties with chemical recyclability. The resulting thermosets and fiber-reinforced composites overcome the traditional trade-off between durability and end-of-life recovery, demonstrating a viable route toward circular thermoset materials.

2. The acetal resin described in this work combines renewable feedstocks, water based chemistry, high material performance, and chemical depolymerization. The cured materials and composites exhibit high stiffness and strength while remaining chemically depolymerizable under mild acidic conditions, enabling recovery of the original building blocks and the composite fibers. This closed-loop approach reduces reliance on fossil resources and minimizes material waste.

3. Further improvements could focus on improving reaction efficiency such as by reducing curing temperatures or reaction times. This proof of concept study could be expanded by introduction of a wider library of biobased and sustainable alcohols and aldehydes.

## Introduction

Thermosetting polymers are indispensable in modern material science and industry, owing to their exceptional strength, rigidity, chemical resistance, and thermal stability.^1^ Unlike thermoplastics, which soften and can be reshaped upon heating, thermosets undergo irreversible covalent crosslinking during curing. This permanent three-dimensional network imparts outstanding dimensional stability under harsh conditions, making thermosets the material of choice in demanding applications ranging from aerospace and automotive composites to protective coatings, adhesives, and electronic encapsulants.^1^ However, these same characteristics that provide durability also create major challenges at the end of life. Conventional thermosets cannot be reprocessed, reshaped, or chemically recycled, and their waste is typically incinerated or landfilled, leading to environmental pollution and the loss of valuable resources.^2,3^

The challenge is particularly severe for fiber-reinforced composites, which account for a rapidly growing share of thermoset usage. Composites combine lightweight construction with high strength and stiffness, making them essential in wind energy, aviation, and automotive sectors.^4^ Yet, their dual-component structure, comprising of a thermoset matrix and a glass or carbon fiber reinforcement renders recycling exceedingly difficult. Incineration or landfilling not only destroys the polymer matrix but also prevents recovery of the high-value fibers,^5,6^ which are themselves very costly and energy-intensive to produce.^4,7–9^ With global demand for composites continuing to rise, the accumulation of unrecyclable waste necessitates the urgent development of sustainable thermoset resins that combine high performance with closed-loop recyclability, allowing both the matrix and fibers to be recovered and reused.

Efforts to address this challenge have focused on two complementary approaches: the use of renewable feedstocks and the design of recyclable thermoset networks. The first strategy replaces fossil-derived monomers with bio-based alternatives to reduce carbon footprints and dependence on non-renewable resources. Notable examples include thermosets derived from lignin,^10–13^ vegetable oils,^14–16^ sugars,^17–19^ vanillin^16,20^ and furfural.^21^ The second strategy introduces chemical recyclability, often by incorporating cleavable or dynamic covalent bonds into the network structure. Various dynamic or degradable linkages have been explored, such as imine,^22,23^ disulfide,^24^ Diels–Alder,^25^ ester^26^ and acetal linkages,^27,28^ enabling reprocessing, reshaping, or even full depolymerization of the thermosets under controlled conditions. Despite significant progress, these systems still face major limitations: many rely on fossil-based monomers, involve complex synthetic routes, require toxic reagents or solvents, and often exhibit mechanical and thermal properties inferior to conventional thermosets, restricting their industrial applicability.

Acetal linkages offer a particularly promising pathway for sustainable thermoset design. They are stable under neutral and basic conditions yet undergo rapid and selective hydrolysis in acidic environments, providing a means to combine long-term stability with controlled degradability under specific conditions.^28–32^ Previous studies have demonstrated the feasibility of acetal-containing thermosets based on aromatic aldehydes such as vanillin or furfural.^33,34^ However, these systems are constrained by limited bio-based content, multistep monomer synthesis, solvent use, and poor mechanical properties.

A further challenge in acetal thermoset synthesis is the use of conventional acid catalysts, which remain trapped within the network after curing, promoting gradual hydrolysis during the service life of the material. To address this issue, we recently demonstrated the use of oxalic acid as a decomposable catalyst for the synthesis of hydrolytically stable acetal thermosets from a vanillin-derived aldehyde and diols.^35^ Oxalic acid efficiently catalyzed acetal formation at moderate temperatures and subsequently decomposed during a high-temperature post-curing step, yielding a catalyst-free network with excellent long-term stability. While this strategy successfully addressed the poor stability of acetal thermosets, it still relied on a synthetically demanding vanillin-derived monomer, requiring multiple reaction and purification steps. Moreover, network formation proceeded slowly, requiring more than five days to reach full conversion, thereby limiting practical applicability and scalability. More recently, significantly shorter curing times have been achieved through transacetalization of acetal monomers with diols.^36^ However, this approach still depends on the prior synthesis and purification of the acetal monomers, adding synthetic complexity and reducing the overall sustainability of the process.

To overcome the limited biobased content and need for complex monomer synthesis, the use of simple, commercially available bio-based polyols and aldehydes offers a compelling alternative. Sorbitol, produced by the catalytic hydrogenation of glucose, is an abundant and low-cost polyol with global production exceeding two million tons per year.^37^ Glutaraldehyde, potentially produced from the oxidation of biomass-derived feedstocks,^38^ is perfectly suited to serve as a complementary dialdehyde. Their direct condensation in water provides a straightforward route to densely crosslinked polyacetals without the need for elaborate monomer synthesis, organic solvents, or extensive purification procedures.

**Fig. 1:**
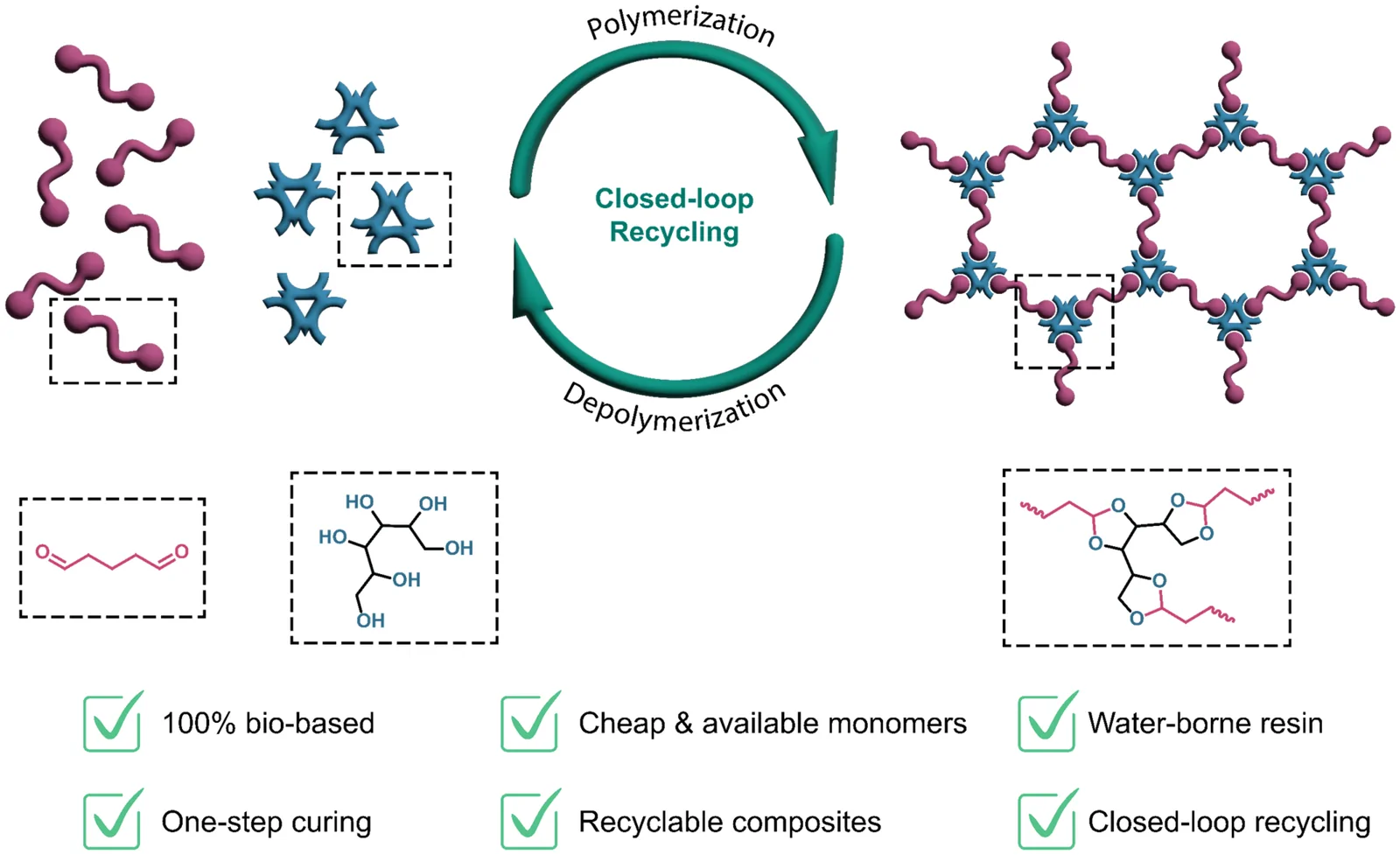
Schematic illustration of the synthesis of a closed-loop recyclable acetal thermoset (idealized structure shown) formed by the condensation of glutaraldehyde and sorbitol. The reaction yields a crosslinked network incorporating acid-labile cyclic acetal linkages, enabling controlled depolymerization and repolymerization into new thermosets.

Herein, we report a new class of fully bio-based, waterborne acetal thermosets synthesized directly from sorbitol and glutaraldehyde using oxalic acid as a green, decomposable catalyst (Fig. 1). In contrast to previous acetal thermosets, the resin is prepared through a simple one-pot aqueous polycondensation and cures within 24 h. The resulting materials combine high bio-based content, hydrolytic stability, excellent mechanical properties, and chemical recyclability. Under acidic conditions, the thermosets undergo complete depolymerization, and the resulting degradation mixture can be directly reused to produce new thermosets with identical properties. Furthermore, the resin exhibits excellent compatibility with both glass and carbon fibers, enabling the fabrication of high-performance recyclable composites from which pristine reinforcement fibers can be readily recovered.

## Results and discussion

### Acetal thermoset preparation

**Fig. 2:**
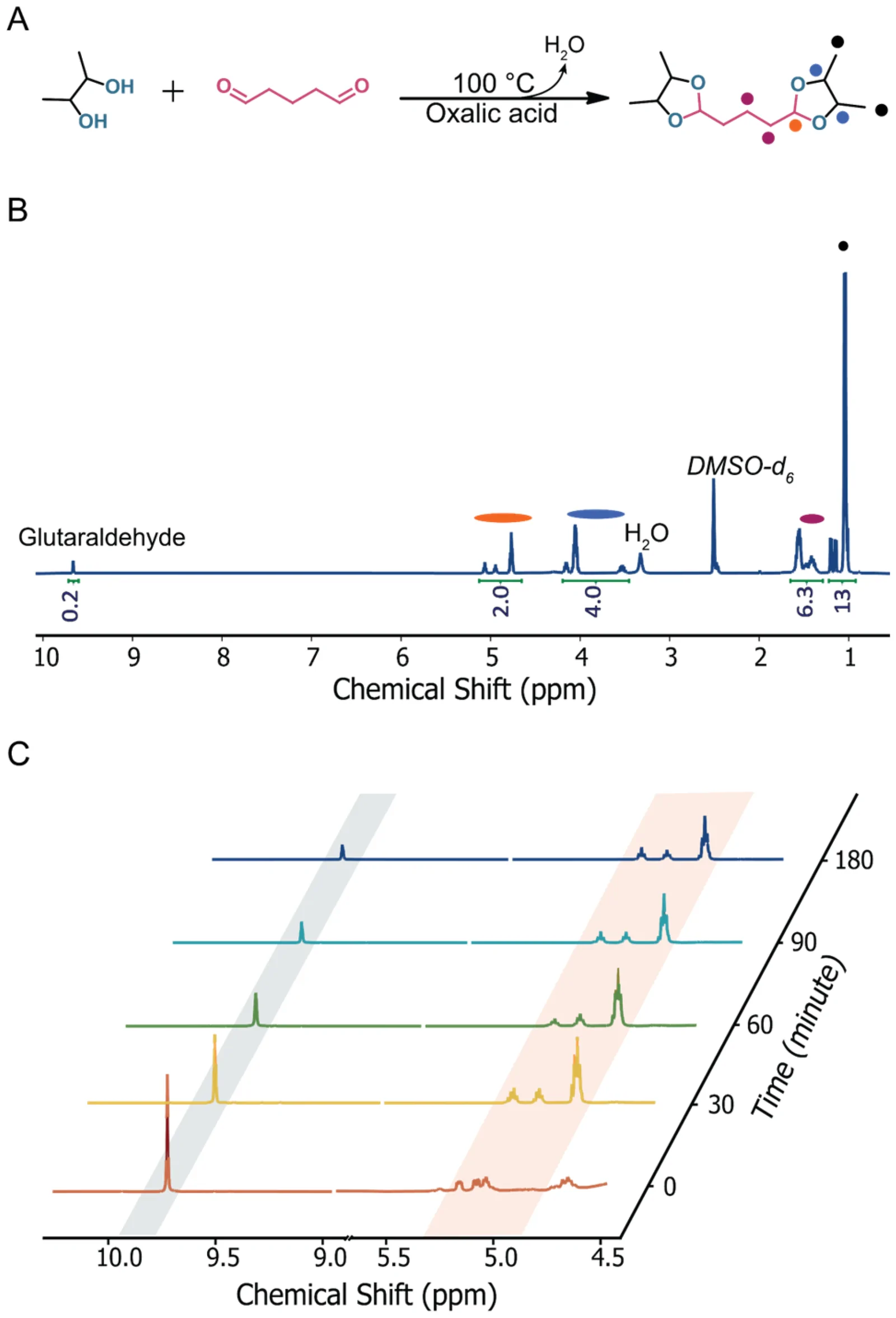
(A) Reaction scheme for the acid-catalyzed condensation of glutaraldehyde with 2,3-butanediol to form the cyclic bis-acetal M1. (B) ^1^H NMR spectrum of the final reaction mixture after 3 hours, confirming the formation of M1. (C) Stacked ^1^H NMR spectra illustrating the evolution of the reaction, highlighting the decrease of the aldehyde proton signal at 9.7 ppm and the concurrent appearance of characteristic cyclic acetal signals in the 4.7–5.2 ppm region (DMSO-d_6_, 400 MHz).

To elucidate the chemical structure resulting from the condensation of sorbitol with glutaraldehyde, as well as the associated reaction kinetics, a model reaction was employed. Small-molecule analogues bearing functional groups that closely resemble those of the polyfunctional reactants were used with oxalic acid as an acid catalyst, enabling accurate analysis of both the reaction product and kinetics of the reaction (Fig. 2, Schemes S1 and S2). The reaction progress was monitored by ^1^H NMR spectroscopy and liquid chromatography-mass spectrometry (LC-MS). The reaction of 2,3-butanediol with glutaraldehyde in the presence of catalytic oxalic acid at 100 °C yielded a bis-acetal molecule (M1) as the major product (see Experimental section for reaction details, Fig. 2A). ^1^H NMR spectroscopy revealed characteristic signals of cyclic acetal protons in the range of 4.7–5.2 ppm, accompanied by a pronounced decrease in the aldehyde proton signal at 9.7 ppm (Fig. 2B), confirming efficient acetal formation. After 3 h, near-complete conversion was achieved, and the product was obtained as a clear, low-viscosity liquid, consistent with the formation of small cyclic acetal species. The preferential formation of cyclic acetals is attributed to the vicinal diol motif of 2,3-butanediol, which favors intramolecular cyclization into thermodynamically stable five-membered rings.^39^

The reaction kinetics were monitored over time by time-resolved ^1^H NMR spectroscopy (Fig. 2C). A rapid initial conversion was observed, exceeding 75% within the first 30 min, indicating that acetal formation proceeds readily under these conditions despite the presence of water. Following the initial fast acetal formation, the reaction rate decreased, approaching equilibrium at 90% conversion after approximately 3 h. A small fraction of residual aldehyde remained, which is attributed to the partial co-evaporation of the diol with water during the reaction, leading to a slight excess of the aldehyde. LC-MS analysis further verified M1 as the dominant product, displaying a major ion at *m*/*z* = 245.33 ([M1 + H]^+^), without the presence of any oligomeric species (Fig. S1).

Having established the preferential and rapid formation of cyclic acetals in the model system, crosslinked polymer networks containing such cyclic acetal motifs were subsequently prepared. An acetal thermoset (AT) was synthesized by mixing sorbitol and aqueous glutaraldehyde (50 wt%) in a 2 : 3 molar ratio with 0.11 wt% oxalic acid as catalyst (see Experimental section for details, Scheme S1). Heating the mixture at 100 °C in a closed vessel produced a homogeneous resin solution, which was cast into films under nitrogen. After curing for 16 h at 100 °C, the material underwent post-curing at 150 °C for 8 h to decompose the oxalic acid catalyst, yielding a stable, catalyst-free network. The infrared (FT-IR) spectrum of the cured film displayed a pronounced band corresponding to the cyclic O–C–O bond (950–1150 cm^−1^), confirming the successful formation of cyclic acetal linkages (Fig. S2). Residual bands were assigned to unreacted hydroxyl (3000–3600 cm^−1^) and aldehyde (1650–1800 cm^−1^) functionalities, which are attributed to kinetic limitations imposed by the high crosslink density, restricting complete conversion.

The network integrity and crosslink density were evaluated through swelling and gel fraction experiments in a variety of solvents, including ethanol, DMF, THF, chloroform, toluene, hexane and water. In all cases, the gel fraction exceeded 95% after 4 days of immersion (Fig. S3), confirming the high degree of crosslinking and excellent solvent resistance. The material swelled most in DMF (31%), while nonpolar solvents such as toluene and hexane caused negligible swelling (Table S1). The measured static water contact angle of 64° (Fig. S4) is comparable to conventional epoxy thermosets,^40–42^ indicating a moderately polar surface well-suited for incorporation of both glass and carbon fibers in the acetal thermoset matrix forming fiber reinforced composites.

### Thermal and mechanical properties of the acetal thermoset

**Fig. 3:**
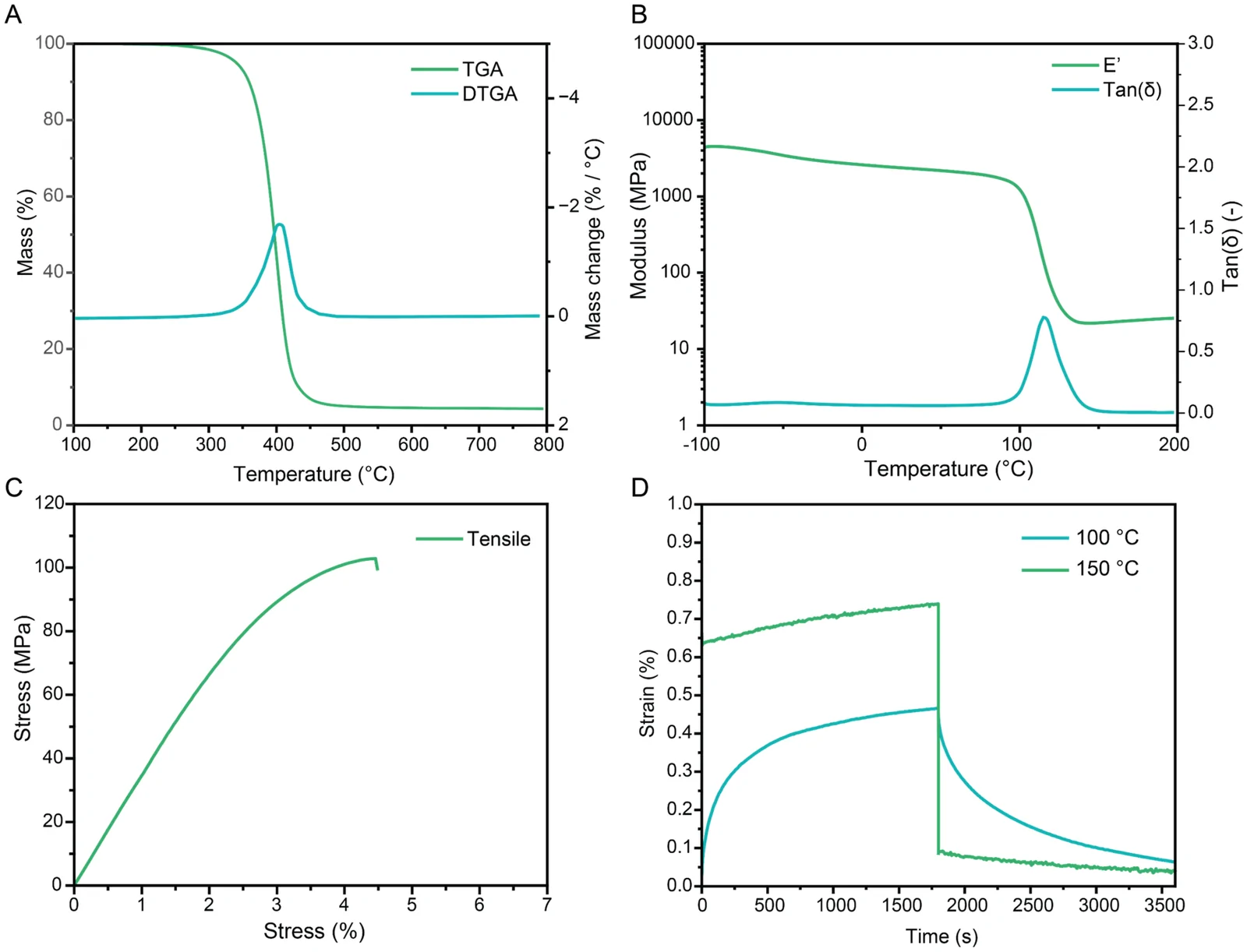
Thermal and mechanical properties of AT. (A) TGA thermogram under nitrogen; (B) DMA showing storage modulus and tan *δ*; (C) tensile stress–strain curve; and (D) constant-stress creep response at elevated temperatures.

Thermal stability was evaluated using thermogravimetric analysis (TGA) under nitrogen (Fig. 3A). AT exhibited a single degradation step with a 5% weight-loss temperature (*T*_d5%_) of 341 °C, surpassing most reported recyclable or bio-based thermosets.^43–47^ The low char residue of less than 5% is expected due to the purely aliphatic nature and the high oxygen content of the network.^43^ Differential scanning calorimetry (DSC) revealed a glass transition at 96 °C with no crystallization or melting transitions (Fig. S5). Dynamic mechanical analysis (DMA) corroborated these findings, displaying a tan *δ* maximum at 114 °C and a smooth transition from the glassy to the rubbery state (Fig. 3B). Notably, a modest increase in modulus across the rubbery plateau was observed, this entropic stiffening behavior indicates a uniform and well-developed crosslinked network.^48^

Tensile testing confirmed that the condensation reaction afforded a well-developed thermoset network displaying outstanding mechanical properties, with an ultimate strength of 99 MPa, a Young's modulus of 3.3 GPa, and an elongation at break of 4.9% (Fig. 3C and Table S2). These properties rival commercial epoxy thermosets and surpass most recyclable or bio-based alternatives reported to date.^12,33,34,44,46,49–54^

Creep and stress-relaxation measurements further demonstrated exceptional network stability. Under constant stress at 100 and 150 °C, AT exhibited minimal deformation and nearly full shape recovery after unloading (Fig. 3D). The slower relaxation near 100 °C arises from the proximity to the *T*_g_, while faster recovery above *T*_g_ reflects increased elastic network mobility. Stress-relaxation tests showed extremely slow relaxation kinetics; even at 230 °C, the time to reach 1/*e* of the initial modulus was approximately 2200 s (Fig. S6). This slow relaxation is attributed to the cyclic acetal structure, high crosslink density, and the absence of residual catalyst, which all limit dynamic bond exchange. These characteristics distinguish AT from previously reported acetal-based dynamic networks,^27,55–57^ including spiro or cyclic acetal motifs which display higher dynamicity and creep.^34,58–60^ Dimensional stability is a critical design criterion for thermosets, thus the high creep resistance and low dynamicity at elevated temperatures highlights the industrial applicability of AT.^61^

The hydrolytic stability of AT, another crucial requirement for practical applications, was evaluated by immersing tensile bars in water for 5 days. Post-immersion specimens (AT-W) showed no detectable swelling, deterioration or loss of shape (Fig. S7A), furthermore no chemical degradation was observed. FTIR spectra displayed unchanged intensity of the aldehyde, OH stretch or acetal bands at 3100–3700 cm^−1^, 1650–1800 cm^−1^ and 1150–950 cm^−1^, respectively (Fig. S7B). The DSC thermogram of AT-W exhibited no significant deviation from AT with only a minor shift in *T*_g_ from 96 to 93 °C (Fig. S7C). Likewise, the mechanical properties remained identical compared to the original samples (Fig. S7D), as summarized in Table S2. These results confirm that AT maintains structural integrity and mechanical robustness after prolonged water exposure.

### Depolymerization and recycling of AT

AT proved to be highly stable under neutral conditions, and yet the acetal linkages enable selective, stimulus-responsive cleavage of the network under acidic environments, motivating a detailed investigation into the recyclability of AT. Immersion of AT fragments in 2 M HCl at 80 °C resulted in complete dissolution within 4 h, while milder acids such as 2 M H_3_PO_4_ required over 48 h for a fully homogeneous solution to form (see Experimental section for reaction details). Consistent with previous reports,^36,62^ ethanol was found to markedly accelerate depolymerization through a *trans*-acetalization mechanism, in which ethanol inserts into the acetal bridge to form soluble ethoxy-terminated oligomers. Consequently, 2 M ethanolic H_3_PO_4_ proved highly effective, enabling full depolymerization within 12 h.

**Fig. 4:**
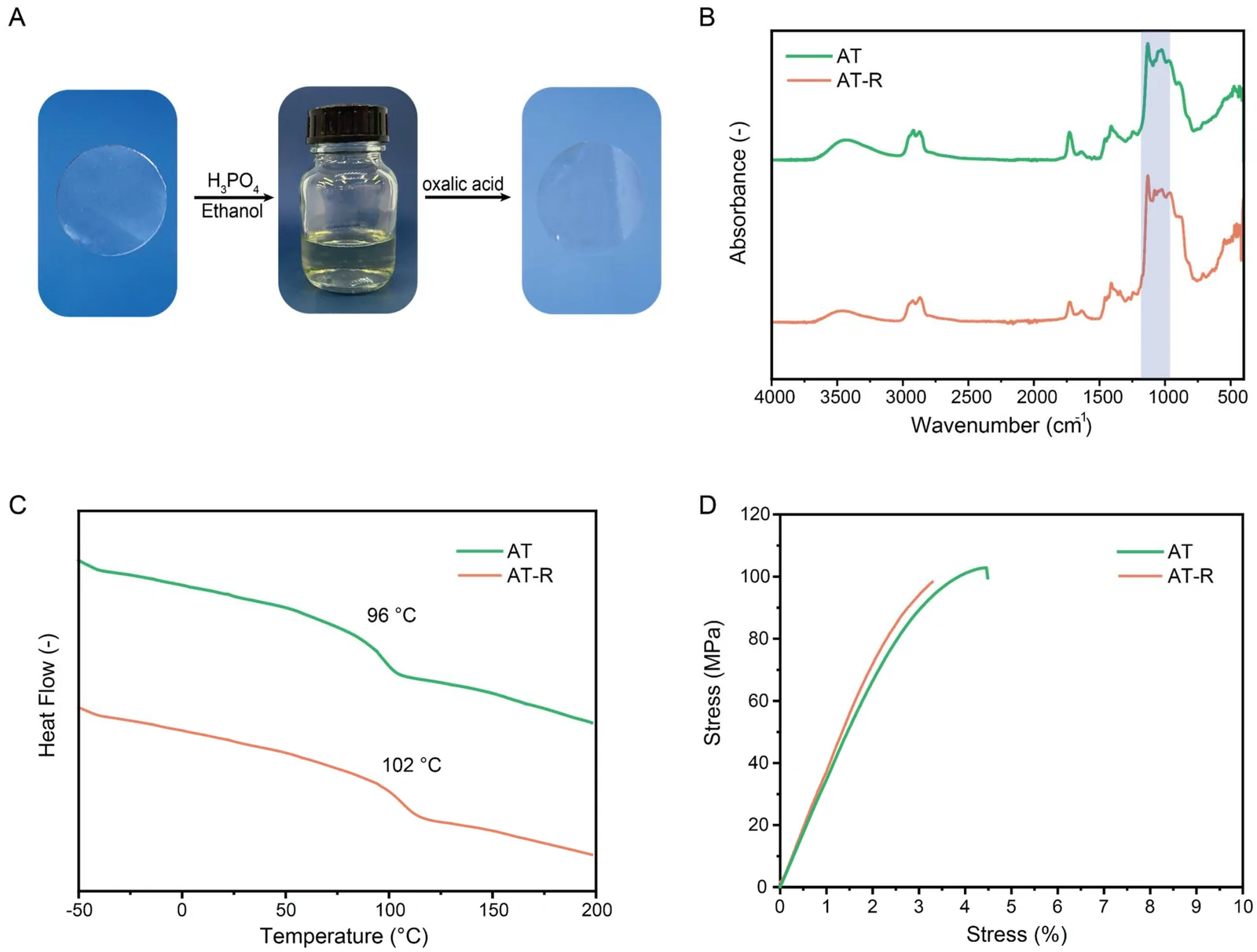
Closed-loop recycling of AT using a 2 M ethanolic H_3_PO_4_ solution at 80 °C. (A) Photographs of the pristine thermoset (AT), the depolymerization mixture and the regenerated thermoset (AT-R). (B) FTIR spectra with the characteristic acetal O–C–O region highlighted in blue, (C) DSC thermograms, and (D) tensile stress–strain curves comparing AT and AT-R.

^1^H NMR spectra of the depolymerization solution revealed signals corresponding to sorbitol, acetalized glutaraldehyde, and soluble acetal oligomers, with minimal formation of free aldehyde species (Fig. S8). This assignment is further supported by GPC analysis of the depolymerization mixture, which revealed the mixture is indeed a polydisperse mixture of oligomers with a number average molar mass of 2070 g mol^−1^, confirming that the depolymerization mixture consists predominantly of low-molecular-weight oligomeric species rather than discrete monomers (Fig. S9). Although these species cannot be readily separated into pure monomers, the depolymerization mixture can be directly used for repolymerization into new thermoset materials. After neutralization and filtration of the resulting phosphate salts, the recovered solution was reused for the condensation into a new thermoset (AT-R). Remarkably, once cured the recycled material displayed nearly identical structural and mechanical characteristics to the original AT (Fig. 4). FTIR spectra confirmed an identical functional group profile, and DSC revealed a modest 6 °C increase in the *T*_g_ to 102 °C. Tensile testing analysis yielded identical tensile strength and Young's modulus (Table S3). These results demonstrate that AT can be chemically depolymerized and reformed in a fully closed-loop manner maintaining the original excellent mechanical properties.

### Acetal based composite fabrication

**Fig. 5:**
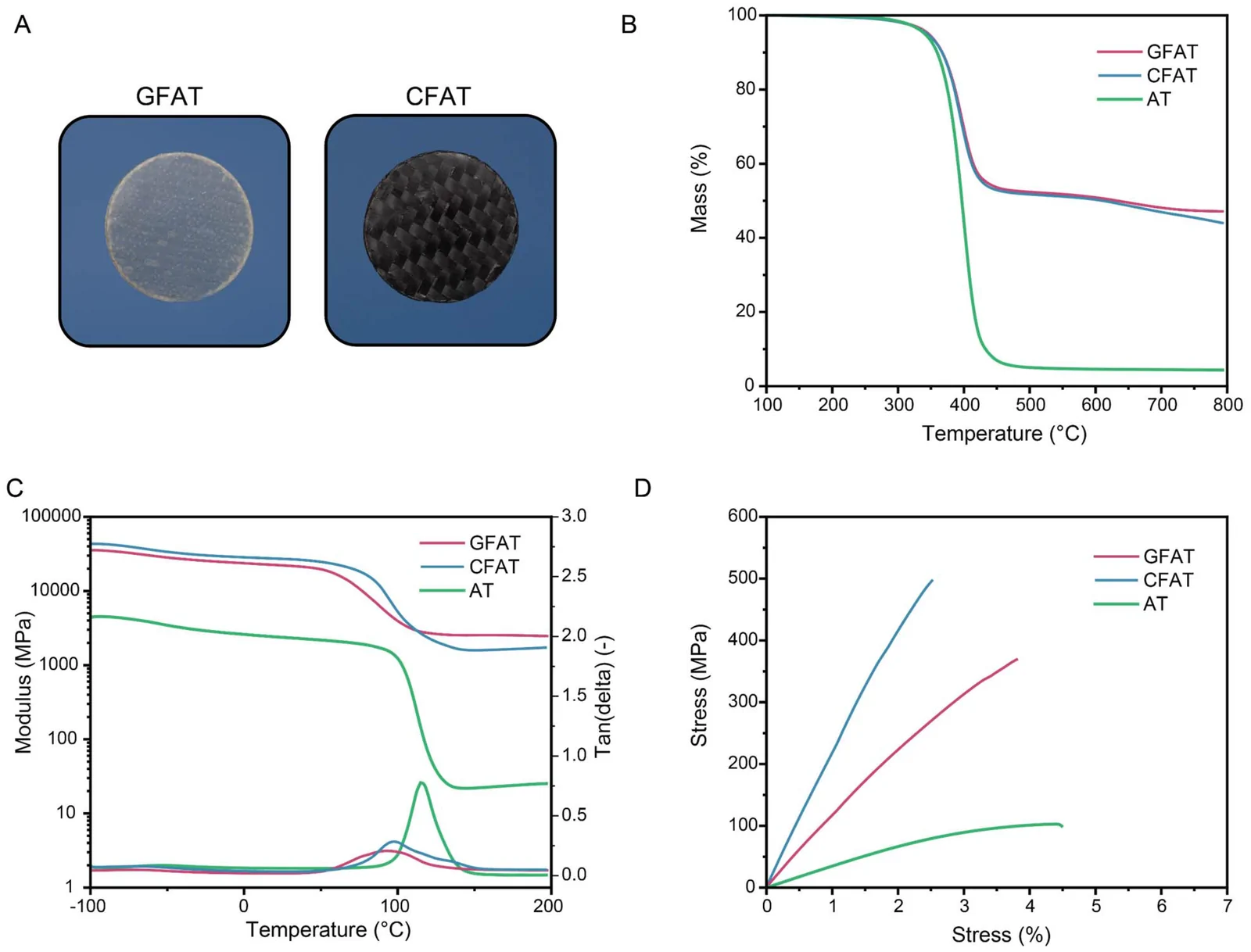
Material properties and characterization of GFAT and CFAT composites. (A) Photographs of the cured thermoset laminates; (B) TGA thermograms, (C) DMA profiles showing storage modulus and tan *δ*, and (D) tensile stress–strain curves of the reinforced composites.

To explore the application of AT as a matrix for structural composites, glass-fiber (GFAT) and carbon-fiber (CFAT) composites were fabricated (see Experimental section for procedural details). Plain-weave glass and carbon fiber fabrics were impregnated by immersion in the waterborne sorbitol–glutaraldehyde resin, manually stacked into laminates, and cured to produce composites containing approximately 50 wt% fiber (Fig. 5A). This straightforward hand lay-up process was deliberately selected to demonstrate the low viscosity, excellent fiber impregnation, and ease of processing afforded by the waterborne acetal resin.

The composites exhibited excellent thermal and mechanical performance. TGA showed *T*_d5%_ values that were comparable to or slightly higher than those of the neat resin with approximately 50% char yield corresponding to the fiber content of the composites (Fig. 5B). DMA revealed *T*_g_ values near 100 °C for both composites (Fig. 5C). In addition, dual cantilever DMA measurements showed room-temperature bending moduli of 4.8 GPa for CFAT and 3.3 GPa for GFAT (Fig. S10). Based on tensile testing CFAT achieved a tensile strength of 510 MPa and tensile modulus of 22.6 GPa, while GFAT reached 390 MPa and 13.1 GPa, respectively (Fig. 5D and Table S4). These results place both composites well within the range of commercial epoxy-based composites.^47^

**Fig. 6:**
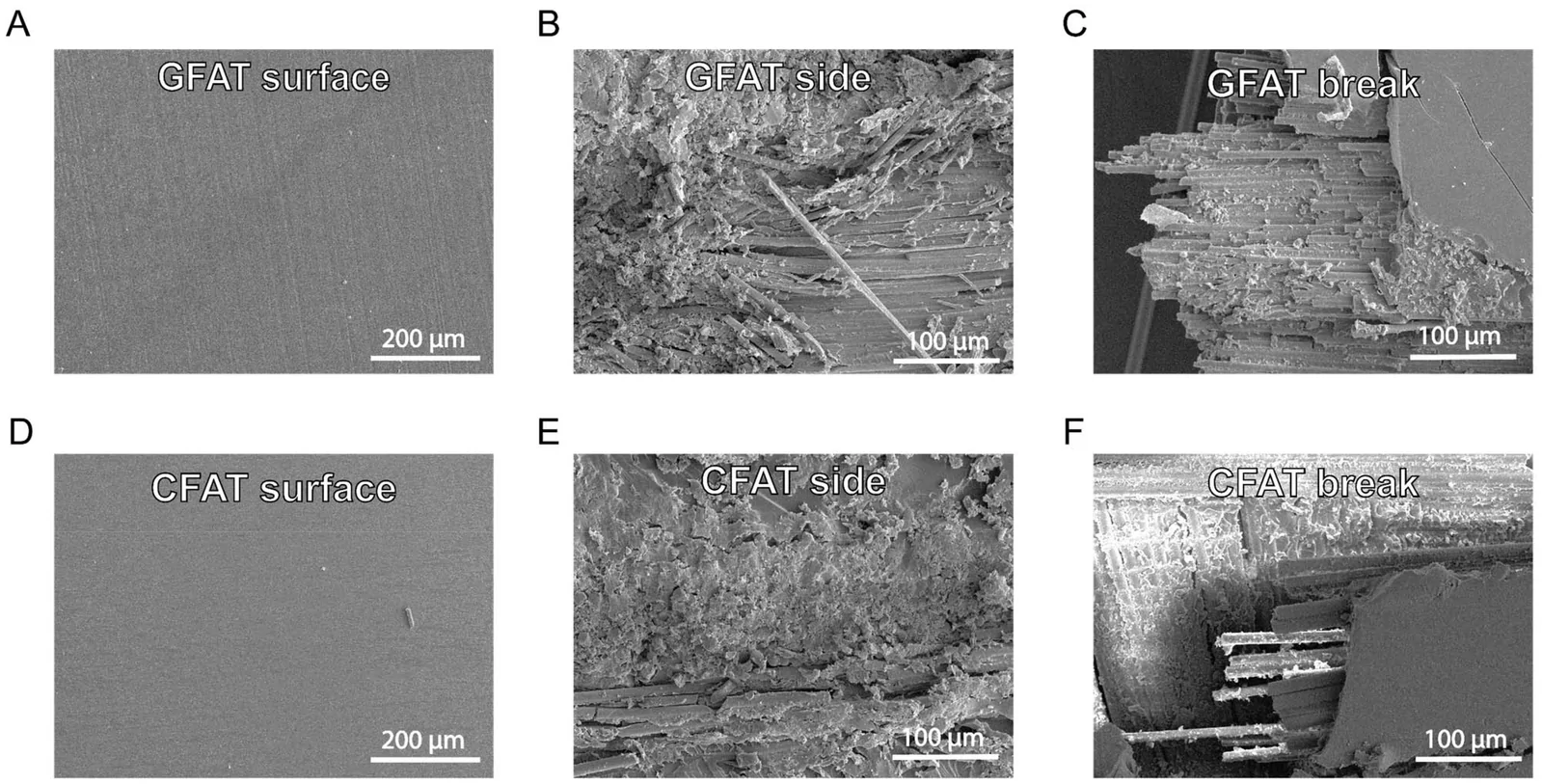
SEM images of GFAT and CFAT showing (A and D) surface morphology of the cured composites, (B and E) cross-sectional images illustrating fiber distribution, and (C and F) fracture surfaces after tensile failure, highlighting good matrix–fiber wetting.

SEM analysis of the composite materials revealed smooth, homogeneous surfaces free from any voids or exposed fibers (Fig. 6A and D). Cross-sectional imaging confirmed uniform fiber wetting and good matrix–fiber adhesion. Imaging of the fracture interface after tensile testing revealed fiber breakage rather than fiber pull-out, with matrix residues adhering to the fiber surface (Fig. 6C and F), indicating efficient interfacial load transfer and strong fiber–matrix bonding.

### Acetal thermoset composite recycling

**Fig. 7:**
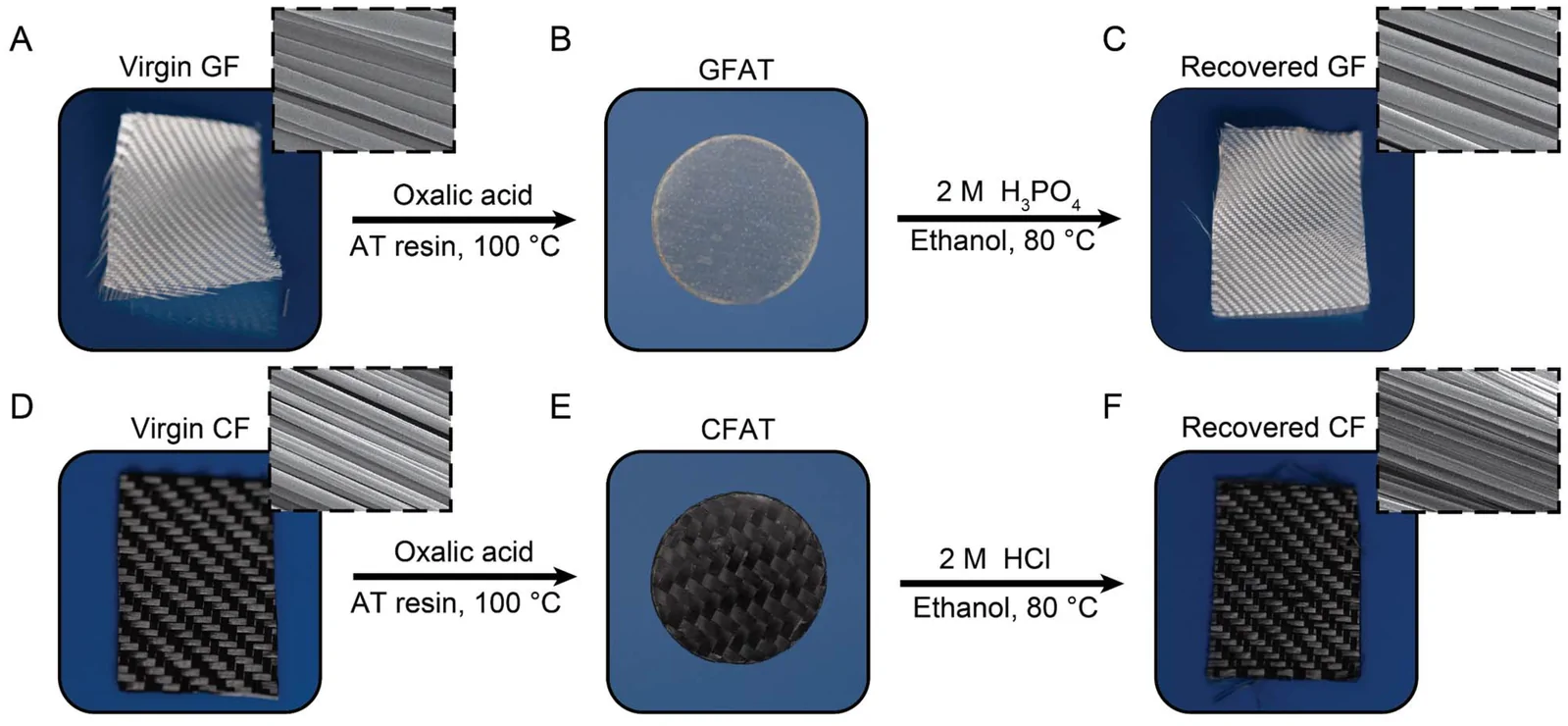
Photographs demonstrating the closed-loop recycling of glass-fiber (GFAT) and carbon-fiber (CFAT) composites. (A and D) Virgin woven fiber fabrics, with insets showing SEM images of the pristine fibers; (B and E) fibers embedded within the cured acetal composite; and (C and F) fully recovered, intact fibers obtained after acid-catalyzed depolymerization of the acetal resin, with insets showing SEM images of the recovered fibres.

Encouraged by the successful depolymerization of the neat resin, the recyclability of GFAT and CFAT composites were investigated. As the fibers are the most expensive and energy intensive component to produce, their recovery is essential from both a waste valorization and environmental impact perspective.^4,7^ GFAT samples were fully depolymerized in 2 M ethanolic H_3_PO_4_ at 80 °C within 15 h, yielding intact, clean glass fibers that were identical in appearance and morphology to virgin fibers (see the Experimental section for details, Fig. 7C and S11B). CFAT composites were significantly more resistant under identical conditions due to the tighter packing, smaller fiber diameter, and the inherent hydrophobic nature of carbon fibers, which collectively limit matrix accessibility to the acidic medium. SEM imaging revealed substantial residual matrix between fibers after 48 h (Fig. S11F). However, complete depolymerization and recovery of pristine carbon fibers was achieved using 2 M ethanolic HCl within 16 h (Fig. 7F and S11E). The recovered fibers were visually and microscopically indistinguishable from the original woven-fiber fabrics. To further emphasize the full recovery of fiber properties, tensile testing of the fiber bundles revealed identical force–strain curves for the recovered fibers compared to the virgin fibers (Fig. S12 and Table S5). These findings demonstrate that both the polymer matrix and reinforcing fibers can be fully recovered through acid-catalyzed depolymerization, establishing a complete closed-loop recycling route for high-performance fiber composites.

## Conclusions

In this work, we report the facile synthesis of a new class of fully bio-based, waterborne acetal thermosets prepared *via* the one-step condensation of sorbitol and glutaraldehyde. Oxalic acid serves as a green, *in situ* decomposable catalyst, promoting efficient acetal network formation while decomposing during post-curing to yield catalyst-free, stable thermosets. The resulting materials exhibit outstanding mechanical properties, with tensile strengths reaching 100 MPa, Young's moduli exceeding 3.3 GPa, and thermal decomposition temperatures above 340 °C, matching or surpassing conventional epoxy resins. The waterborne acetal formulation also serves as an effective matrix for glass- and carbon-fiber composites with excellent mechanical performance. A defining feature of both the neat thermosets and the composites is their deliberately engineered end-of-life recyclability. The incorporation of acid-labile acetal linkages enables selective depolymerization, establishing this system as a rare example of a fully circular, purely acetal-based closed-loop recyclable thermoset. The resulting depolymerization mixture can be directly reused to regenerate thermosets with identical structural and mechanical properties while enabling recovery of pristine reinforcing fibers from composites. Together, these results establish a scalable platform for high-performance, bio-based thermosets that integrates waterborne processing, green catalysis, and true closed-loop recyclability, demonstrating that material performance and circularity can be successfully combined within a single molecular design framework.

## Experimental

### Materials

Glutaraldehyde (50% in water) and 2,3-butanediol (97%) were purchased from TCI Europe. d-Sorbitol (95%) was bought from BLD chemicals. Oxalic acid (99%), 37% HCl solution and phosphoric acid crystals (99%) were procured from Sigma Aldrich. Chloroform-d (99.8%) and dimethylsulfoxide-d_6_ (DMSO-d_6_, 99.9%) were purchased from Cambridge Isotope Laboratories. All chemical compounds were used without further purification, unless stated otherwise. All solvents were ACS Reagent Grade, purchased from Acros Organics, Fisher Scientific or Sigma–Aldrich and used without further purification. Woven glass fiber (Woven glass 2/2 twill 200 g m^−2^) and carbon fiber fabrics (profinish C/F 2/2 twill 210 g m^−2^) were graciously provided by easycomposites.

### Characterization

#### Nuclear magnetic resonance (NMR) experiments

^1^H spectra were recorded in deuterated dimethyl sulfoxide (DMSO-d_6_) on Bruker UltraShield (400 MHz) at room temperature. ^1^H spectra were taken using 32 scans and a relaxation time of 2 s.

#### Liquid chromatography-mass spectrometry (LC-MS)

LC-MS analyses were performed on a Thermo Scientific LTQ linear ion trap mass spectrometer equipped with an electrospray ionization (ESI) source. Chromatographic separation was achieved using a Phenomenex Kinetex EVO C18 column (5 µm, 4.6 × 100 mm). The mobile phase consisted of water (0.1% formic acid, solvent A) and acetonitrile (0.1% formic acid, solvent B), the gradient was held constant at 5% B for 1 min, increased linearly to 100% B over 6 min, and maintained at 100% B for 1 min, followed by re-equilibration to initial conditions. The flow rate was 0.3 mL min^−1^. Mass spectra were acquired in positive ion mode over an *m*/*z* range of 200–1000.

#### Fourier transformation infrared spectroscopy (FTIR)

FTIR was carried out in attenuated total reflection (ATR) mode on a Thermo Scientific NICOLET iS20. 8 scans were performed over the wavenumber range of 4000 to 450 cm^−1^.

#### Differential scanning calorimetry (DSC)

DSC measurements were performed on TA Q2000. 5–10 mg samples were used in an aluminum pan. The experiments were carried out from −80 to 200 °C at a rate of 5 °C min^−1^ under argon atmosphere. Glass transition temperatures (*T*_g_) were determined by taking the midpoint of the reversible endotherm of the 2nd heating.

#### Thermogravimetric analysis (TGA)

TGA analyses were performed using TGA550 (TA instrument). Samples (5–10 mg) were first equilibrated at 100 °C for 30 minutes, followed by heating from 100 to 800 °C with a rate of 10 °C min^−1^ under a nitrogen atmosphere.

#### Dynamic mechanical analysis (DMA)

DMA measurements were performed on DMA850 (TA instrument) using samples with a thickness of approx. 0.3 mm and a width of 4.2 mm. Tensile mode analysis was carried out from −100 °C to 200 °C at a heating rate of 3 °C min^−1^ under an oscillatory strain of 0.5% and a frequency of 1 Hz. Dual cantilever analysis was performed under identical conditions except a strain of 1% was applied. The glass transition temperatures were recorded as the maximum value of tan *δ*. The creep experiments were performed in tensile mode at a constant stress of 200 kPa at 100 and 150 °C, with force applied for 30 minutes and creep recover for an additional 30 minutes.

#### Tensile testing

Tensile tests of the thermosets and composites were performed on Zwick/Roell Intelligent testing with 2.5 KN load cell using dumbbell-shaped specimens (effective length: 18 mm, width: 1.9 mm, and measured thickness around 0.3 mm) with a strain rate of 10 mm min^−1^. The Young's modulus of the materials was determined by calculating the derivative of the stress–strain curves between 0.4 and 0.8%. The Young's moduli and elongation at break reported are the mean values of at least four different samples. For the analysis of the recovered fibers individual carbon fiber bundles were separated from the woven fabric, each bundle had a length of 6 cm and an approximate weight of 11 mg for the glass fibers three 6 cm bundles with a total weight of 13 mg were used per measurement. The strain rate was 5 mm min^−1^, and the initial grip-to-grip distance was 6 mm, values reported are the mean values of three different samples.

#### Contact angle

Static contact angle measurements were conducted on a OCA30 goniometer (Dataphysics), using MilliQ water (resistivity of 18.2 MΩ cm) as probe liquid with a droplet volume of 3 µL and measurements were performed on at least four different spots per sample, recording the contact angle for at least 20 s using the OCA20 software. Values reported are the average of all individual left and right contact angle values after 20 s measurement time.

#### Gel permeation chromatography

GPC measurements of the depolymerization mixture of AT were performed on LC-250C (Shimadzu) equipped with two PSS GRAM liner 10 μm columns and a refractive index (RI) detector. The depolymerization mixture was dissolved in DMF with 0.1 M LiBr (6 mg ml^−1^), and the solution was filtered through a 0.2 μm PTFE filter before analysis. DMF was used as the eluent with a flow rate of 1 ml min^−1^. The GPC traces were calibrated with a polymethyl methacrylate (PMMA) as standard to calculate the molecular weight.

#### Stress relaxation analysis

Stress relaxation analysis was performed on Discovery HR20 (TA instrument) using samples with a thickness of approx. 0.3 mm and a diameter of 8 mm. The relaxation modulus (*G*_(t)_) was followed over different time periods for a constant applied strain of 1% at the constant temperature (230 to 200 °C). The relaxation modulus (*G*) was normalized by initial value (*G*_1_), and the relaxation time (τ*) was defined as the time when 
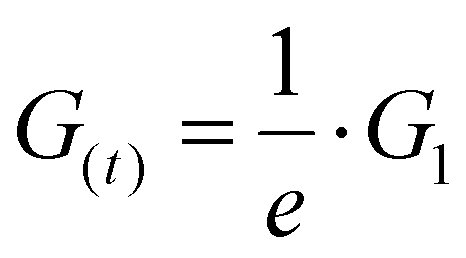
.

#### Scanning electron microscopy (SEM)

Images were obtained using an FEI Quanta 200 3DFEG at an acceleration voltage of 10 kV. Prior to imaging, the samples were sputter-coated with platinum (40 mA, 40 seconds). Top and side view images of the composites and fibers were captured.

#### Swelling and gel content determination

Swelling and gel content experiments were performed by submerging acetal thermosets specimens (100–200 mg) in 10 ml of solvent for four days at room temperature. After removal from the solvent, excess solvent was wiped from the sample surface before weighing. The samples were dried in a vacuum oven at 80 °C for 24 hours, and re-weighed to determine the gel fraction, expressed as the percentage of the remaining weight compared to the initial weight. Solvent resistance, swelling ratio and gel content of the acetal thermoset (AT) were calculated. Dry samples of the polymers (*m*_0_), the weight of the samples after surface drying (*m*_s_) representing the swollen mass, and finally, the sample weight after drying (*m*_d_) were used to calculate the swelling ratio and the gel content using the following two equations:
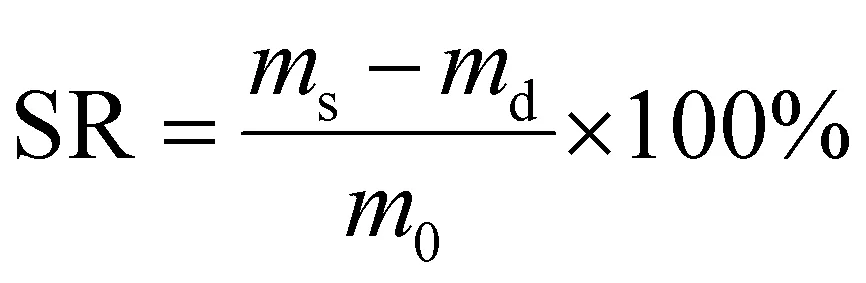

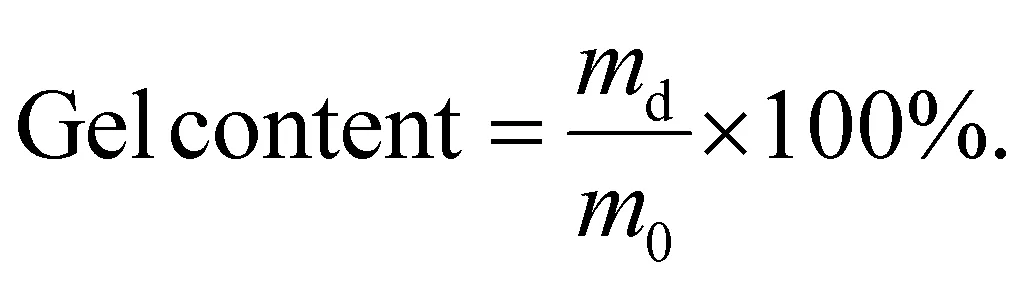


### Experimental procedures

#### Synthesis of acetal compound M1

2,3-Butanediol (17 g, 189 mmol, 2.0 eq.), aqueous glutaraldehyde solution (25 g, 94 mmol, 1.0 eq.), and oxalic acid (0.05 g, 2 mmol, 0.11 wt%) were charged into a 100 mL three-neck round-bottom flask equipped with a short-path distillation head and an argon inlet. The reaction mixture was heated to 100 °C in an oil bath under vigorous stirring and a continuous argon flow. After 3 h, the reaction was allowed to cool to room temperature, yielding a viscous liquid identified as M1.

#### Synthesis of acetal thermoset (AT)

Sorbitol (60 g, 329 mmol, 2 eq.), glutaraldehyde solution (100 g, 500 mmol, 3 eq.) and oxalic acid (0.18 g, 2 mmol, 0.11 wt%) were combined in a closed-capped jar in a nitrogen oven at 100 °C. Once fully transparent and homogeneous, the mixture was then poured in a thin layer on top of aluminum foil placed in the nitrogen oven, maintaining the temperature at 100 °C for 16 h. After this period, the material was cured at 150 °C for 8 hours to remove the oxalic acid. The resulting film AT was separated from the aluminum foil and characterized without further processing.

#### Chemical recycling of acetal thermoset AT

15 g of AT was treated with 100 ml 2 M H_3_PO_4_ ethanolic solution at 80 °C for 6 hours with continuous stirring. The resulting homogeneous solution was neutralized using NaOH ethanolic solution, resulting in the formation of phosphate salts. The ethanolic solution containing acetal oligomers was filtered removing the salt as a white precipitate. After filtration 30 mg (0.2 wt%) oxalic acid was added and once fully dissolved, the mixture was poured in a thin layer on top of aluminum foil placed in a 50 °C nitrogen oven. After 1 hour the temperature was increased to 100 °C for 15 h to fully cure the recycled network. The temperature was increased to 150 °C for 8 h for a final post curing step.

#### Synthesis and characterization of acetal thermoset composites

Sorbitol (30 g, 165 mmol, 2 eq.), glutaraldehyde solution (50 g, 250 mmol, 3 eq.) and oxalic acid (0.09 g, 1 mmol, 0.16 wt%) were combined in a closed-capped jar a nitrogen oven at 100 °C. Once clear and homogeneous, either glass or carbon fiber woven mats are fully submerged and fully saturated with the resin. Composite assemblies were prepared by layering two impregnated fiber sheets on top of each other in the nitrogen oven and firmly pressed the assembly to remove excess resin, maintaining the temperature at 100 °C for 16 h. After this period, the material was cured at 150 °C for 8 hours to decompose the oxalic acid, resulting in carbon fiber and glass fiber acetal thermoset composites (CFAT & GFAT) both containing approximately 50 wt% fiber reinforcement.

### Acetal thermoset composites recycling and fiber recovery

GFAT and CFAT samples were cut in rectangles approximately 3 by 5 cm, and fully submerged in 2 M acid solution at 80 °C without stirring. As a thermoset matrix depolymerized into soluble oligomer the woven fiber sheets were liberated, after 15 hours the fiber sheets were removed and washed with a 1 : 1 ethanol water mixture, and subsequently dried in a 80 °C oven for 4 hours, yielding dried woven fiber sheets identical to the virgin material.

## Conflicts of interest

There are no conflicts to declare.

## Supplementary Material

GC-028-D6GC03576K-s001

## Data Availability

Supplementary information (SI): additional synthesis schemes and characterization methods, including: LC-MS, NMR, FTIR, DSC, TGA, DMA, SEM, contact angle, rheological, chemical stability and tensile testing results. See DOI: https://doi.org/10.1039/d6gc03576k.
