# Supplementary material for: Recyclable and bio-based waterborne acetal thermosets and composites
Source: Green Chem. 2026 Aug 15;28(35):14367–77. doi: 10.1039/d6gc03576k (PMC13496943; doi:10.1039/d6gc03576k)
Supplement: Supplementary file 1 [file GC-028-D6GC03576K-s001.pdf]

This journal is © The Royal Society of Chemistry 2026

Supporting information

Recyclable and Bio-Based Waterborne Acetal Thermosets and Composites

Patrick Schara[1]* and Željko Tomović[1]*

[1] Polymer Performance Materials Group, Department of Chemical Engineering and Chemistry and Institute for Complex Molecular Systems (ICMS), Eindhoven University of Technology, 5600 MB Eindhoven, The Netherlands.
Email: p.j.schara@tue.nl; z.tomovic@tue.nl

Table of Contents

Synthesis of acetal monomer & thermoset (AT) 3

Characterization of acetal thermosets by FTIR spectroscopy 4

Stability, swelling, and gel fraction testing of AT in water and various organic solvents 4

Water-contact angle measurements 5

Chemical recycling of acetal thermosets 7

Synthesis and characterization of acetal thermoset composites 8

Synthesis of acetal monomer & thermoset (AT)

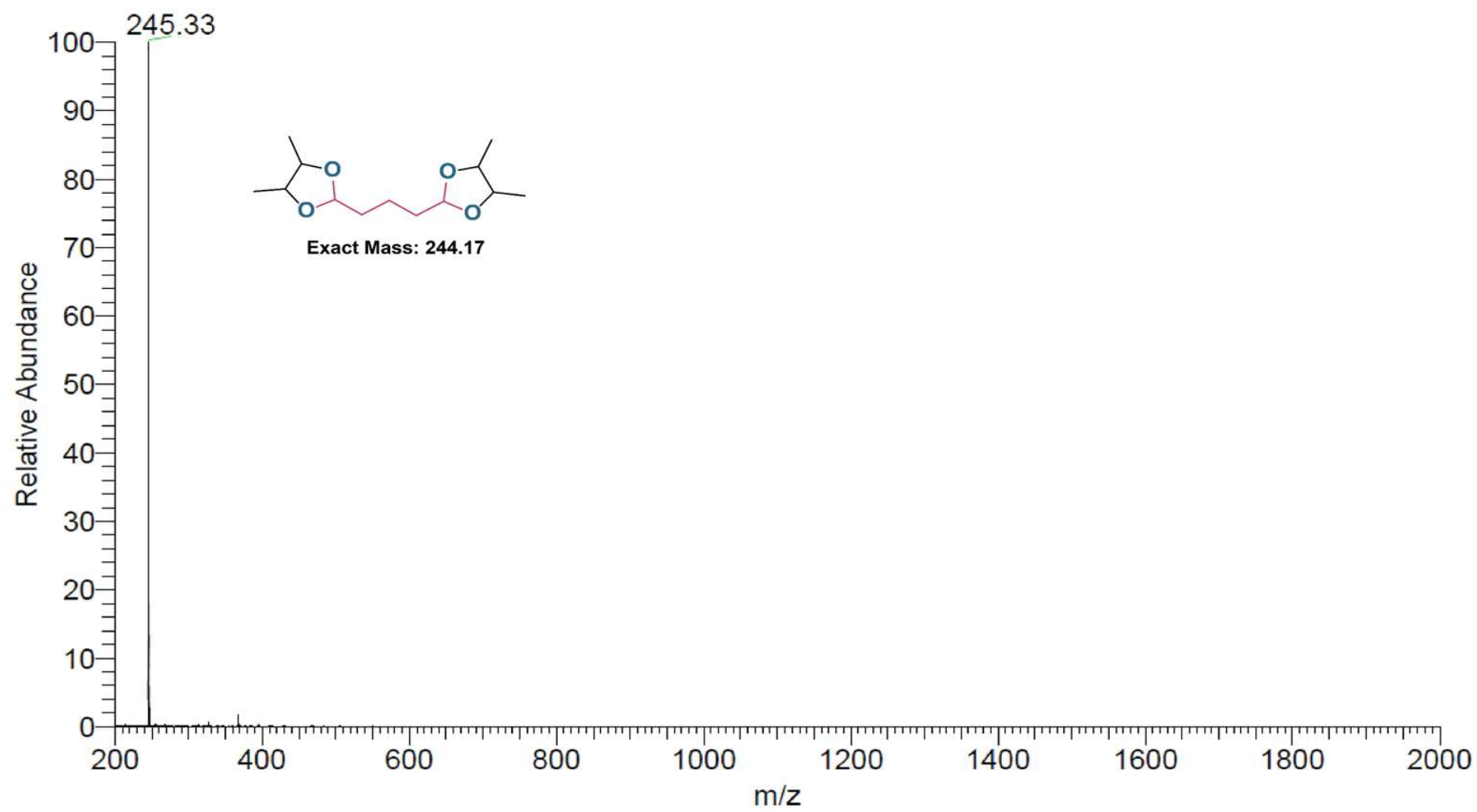

Figure S1: LC–MS spectrum of M1 displaying a single dominant ion corresponding to the expected bis-acetal structure ($M+H^+$)

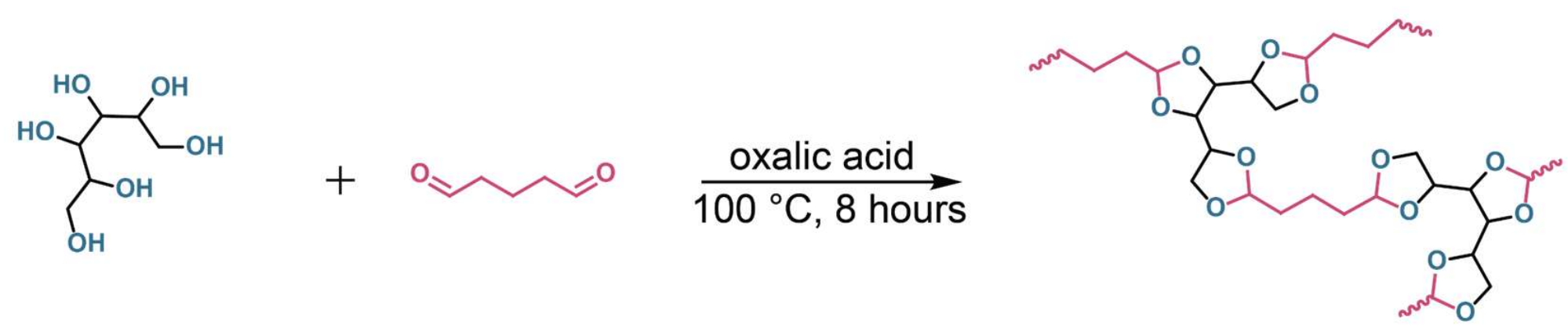

Scheme S1: Polycondensation of sorbitol and glutaraldehyde yielding the acetal thermoset (AT) through formation of cyclic acetal crosslinks.

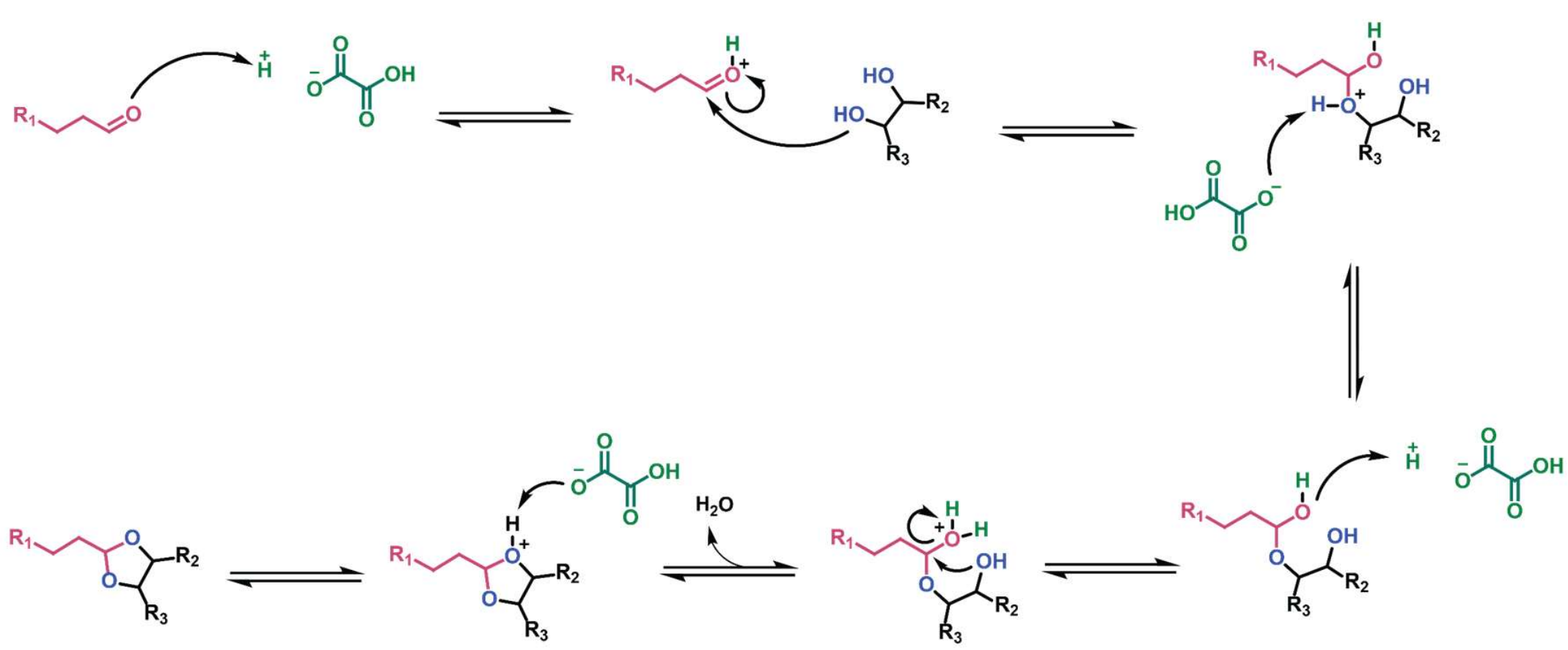

Scheme S2: Proposed oxalic acid-catalyzed mechanism for the formation of cyclic acetals from aldehydes and vicinal diols.

Characterization of acetal thermosets by FTIR spectroscopy

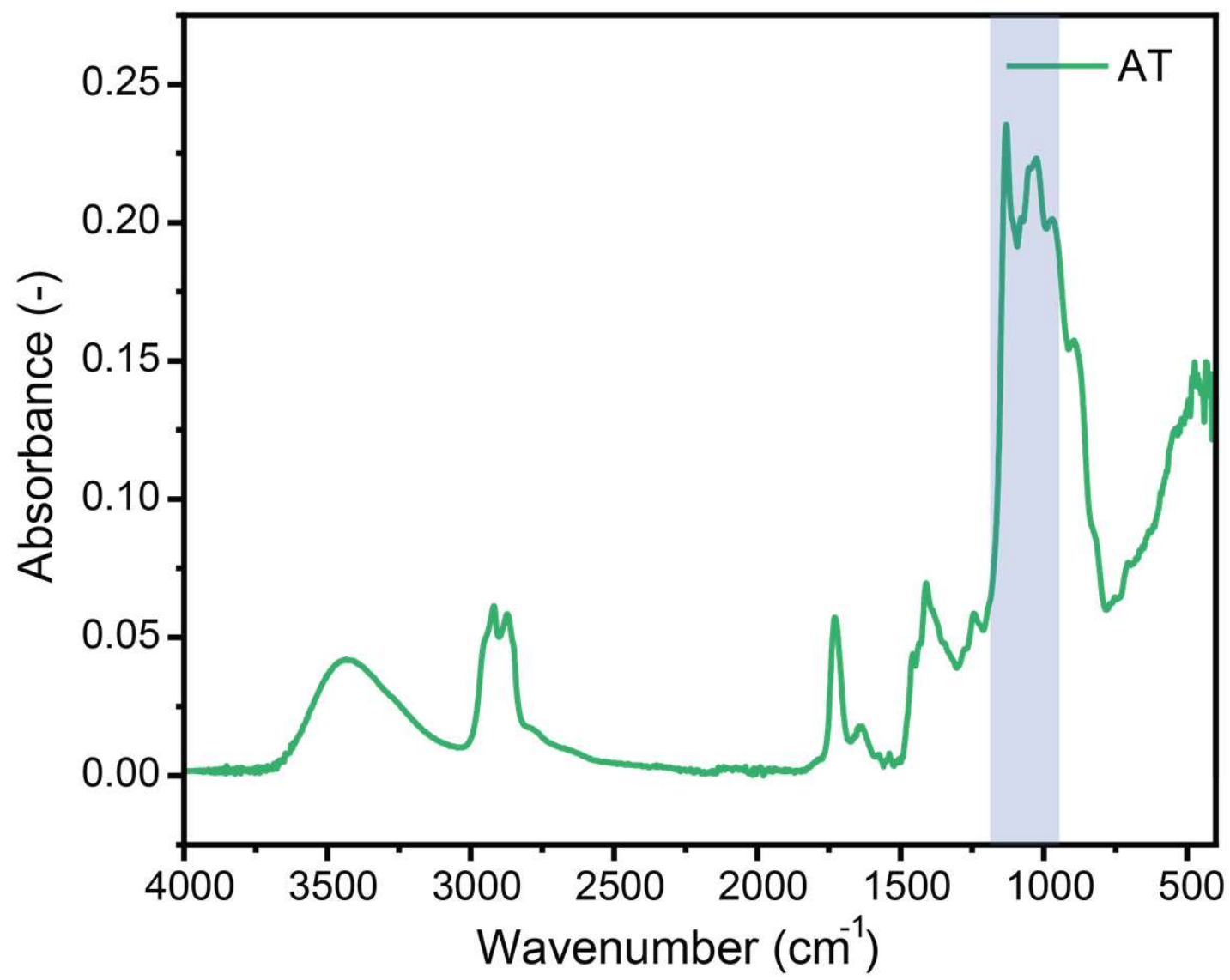

Figure S2: FTIR spectrum of AT, with the characteristic acetal O–C–O region highlighted in blue.

Stability, swelling, and gel fraction testing of AT in water and various organic solvents

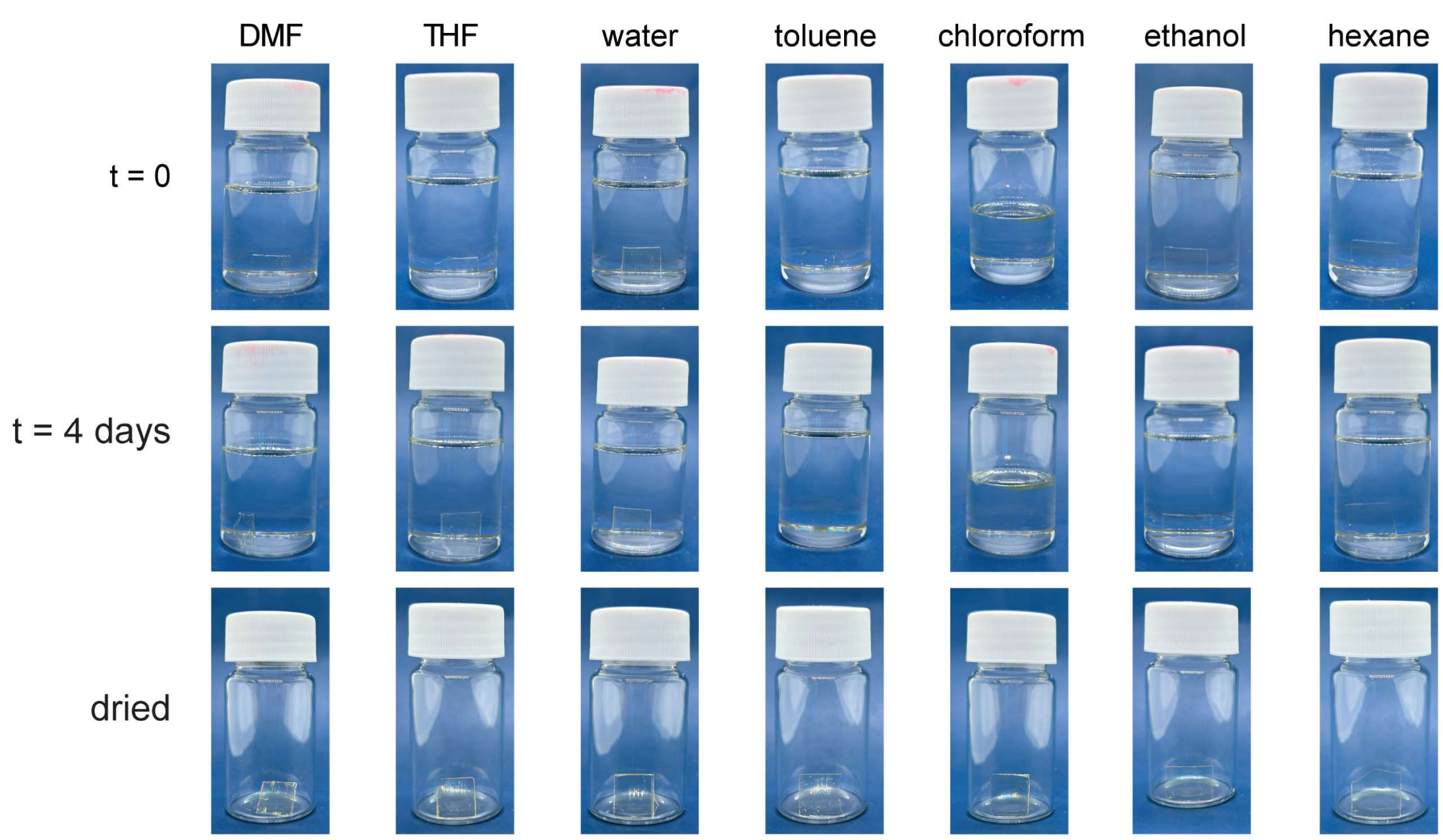

Figure S3: Photographs of AT samples before and after 4-day immersion in various solvents (DMF, THF, water, toluene, chloroform, ethanol, and hexane), demonstrating excellent solvent resistance.

Table S1: Swelling measurements of AT in different solvents at room temperature

Solvent	DMF	THF	Water	Toluene	Chloroform	Ethanol	Hexane
Swelling Ratio (%)	31.2	16.0	4.4	0.4	10.4	0.2	0
Gel Content (%)	99.6	99.1	97.8	96.8	99.1	98.1	98.5

Water-contact angle measurements

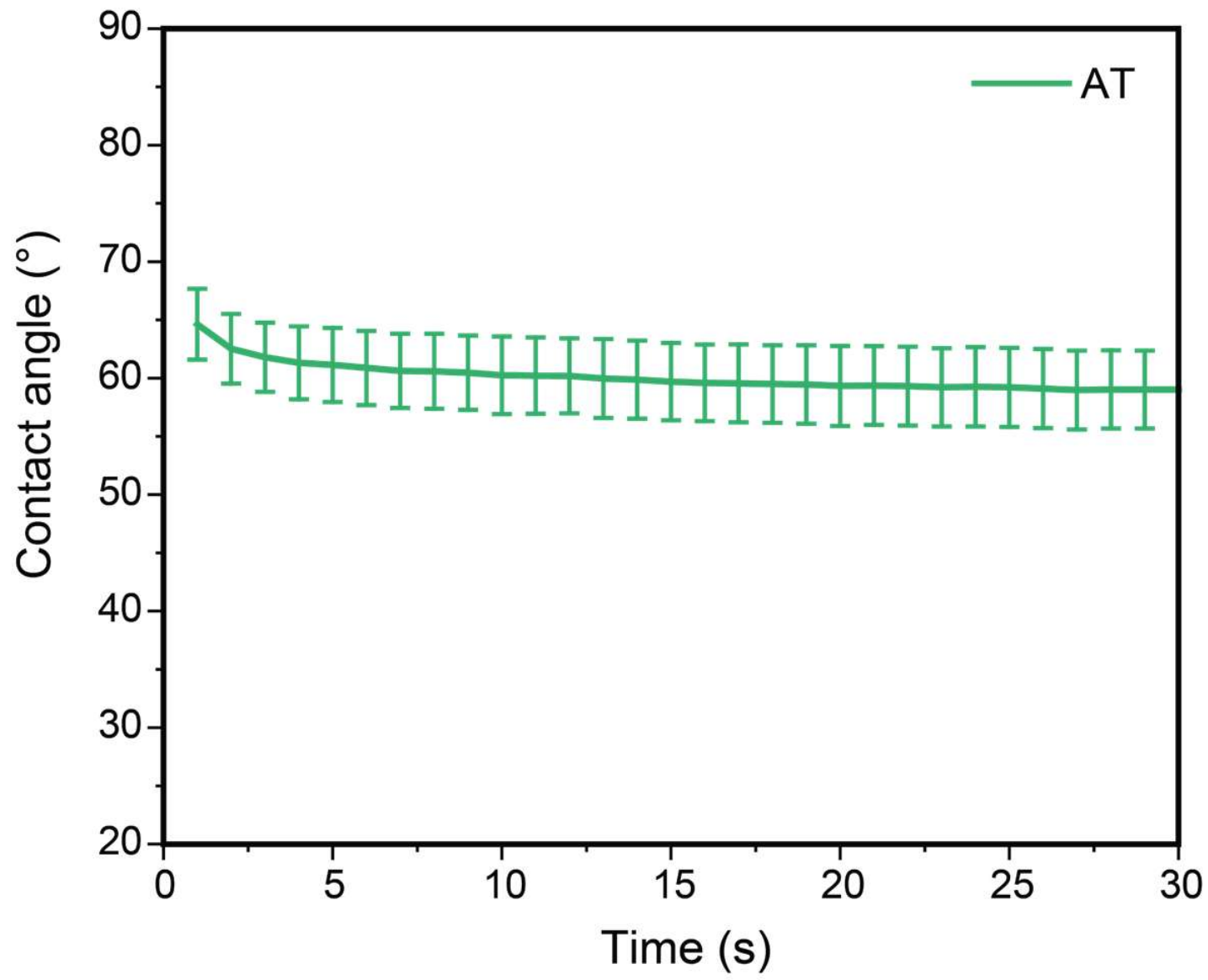

Figure S4: Static water-contact-angle measurements of AT recorded over 30 seconds.

2.3 Thermal and Mechanical Properties

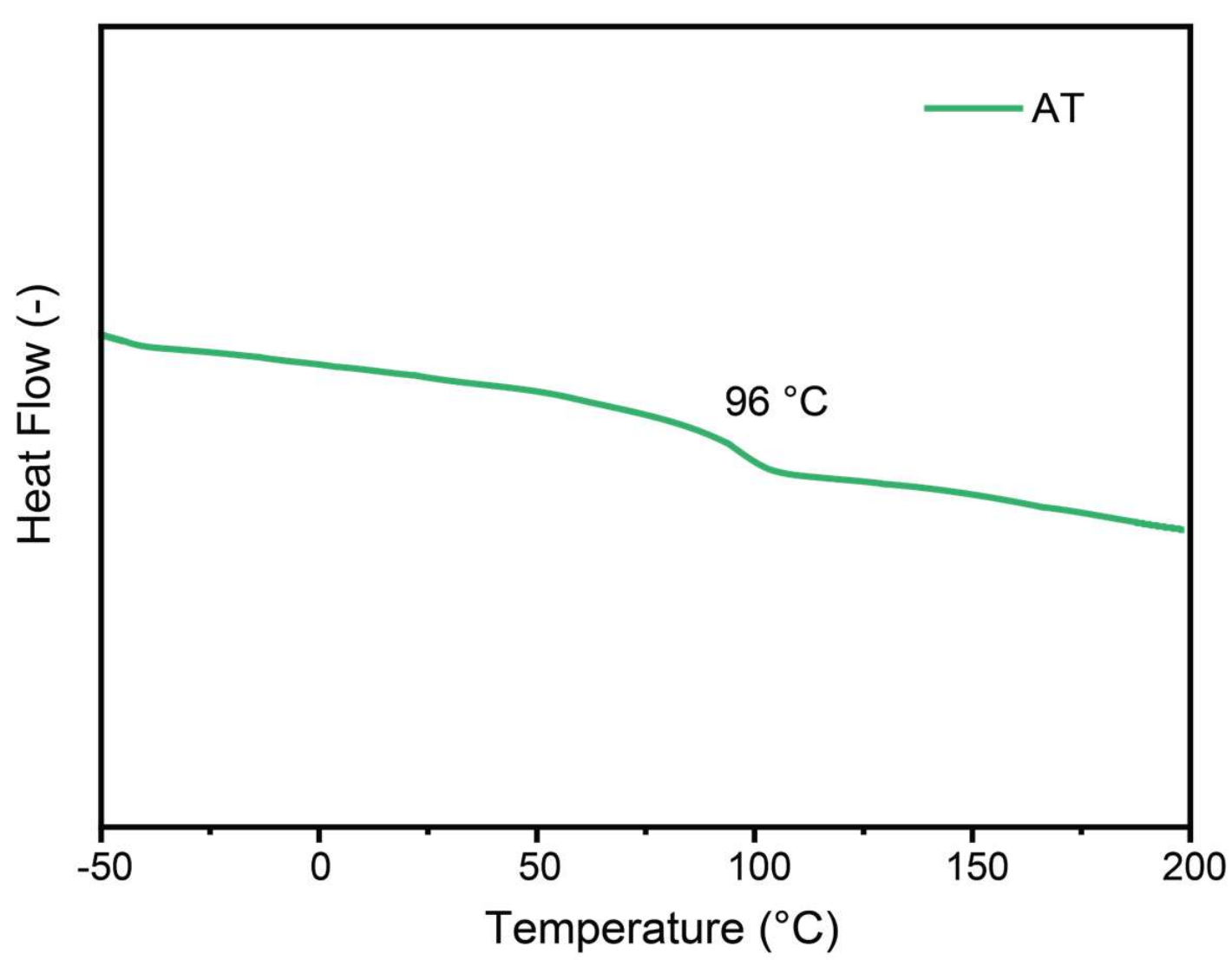

Figure S5: DSC thermogram of AT showing a well-defined glass transition at 96 °C without additional thermal transitions.

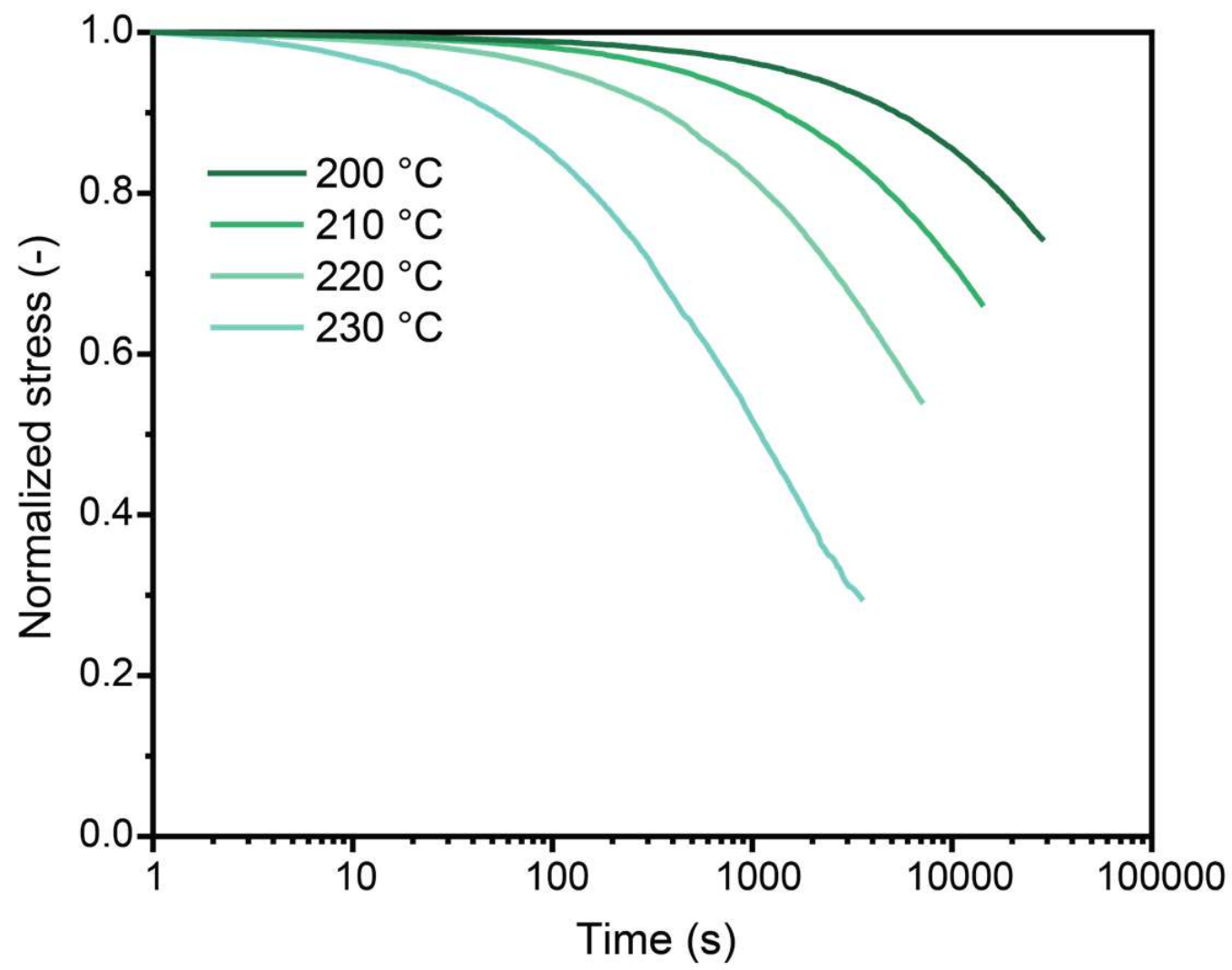

Figure S6: Normalized stress-relaxation curves of AT measured between 230 and 200 °C, demonstrating extremely slow relaxation and high network stability even at elevated temperatures.

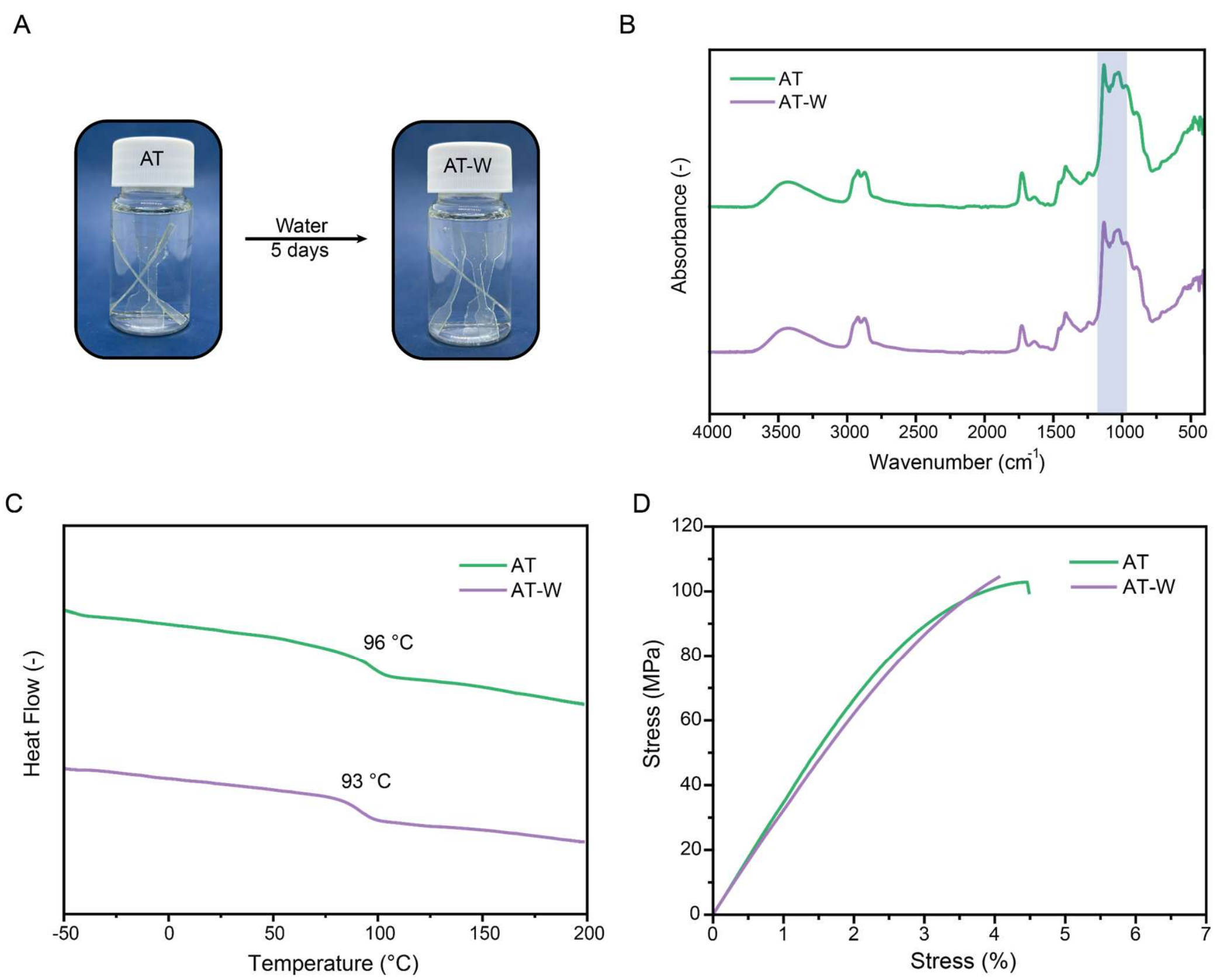

Figure S7: Hydrolytic stability evaluation of AT following 5-day water immersion: (A) photographs of samples before and after immersion, (B) FTIR spectra with the characteristic acetal O–C–O region highlighted in blue, (C) DSC comparison, and (D) tensile testing curves, all confirming preserved structural and mechanical integrity of AT-W.

Table S2: Properties of AT and AT-W based on DSC and tensile testing

Sample	T_g (DSC) (°C)	Elongation at break (%)	Stress at break (MPa)	Young's modulus (GPa)
AT	96	4.9 ± 0.5	98.8 ± 6.6	3.3 ± 0.3
AT-W	93	4.5 ± 0.8	103.4 ± 8.1	3.2 ± 0.2

Chemical recycling of acetal thermosets

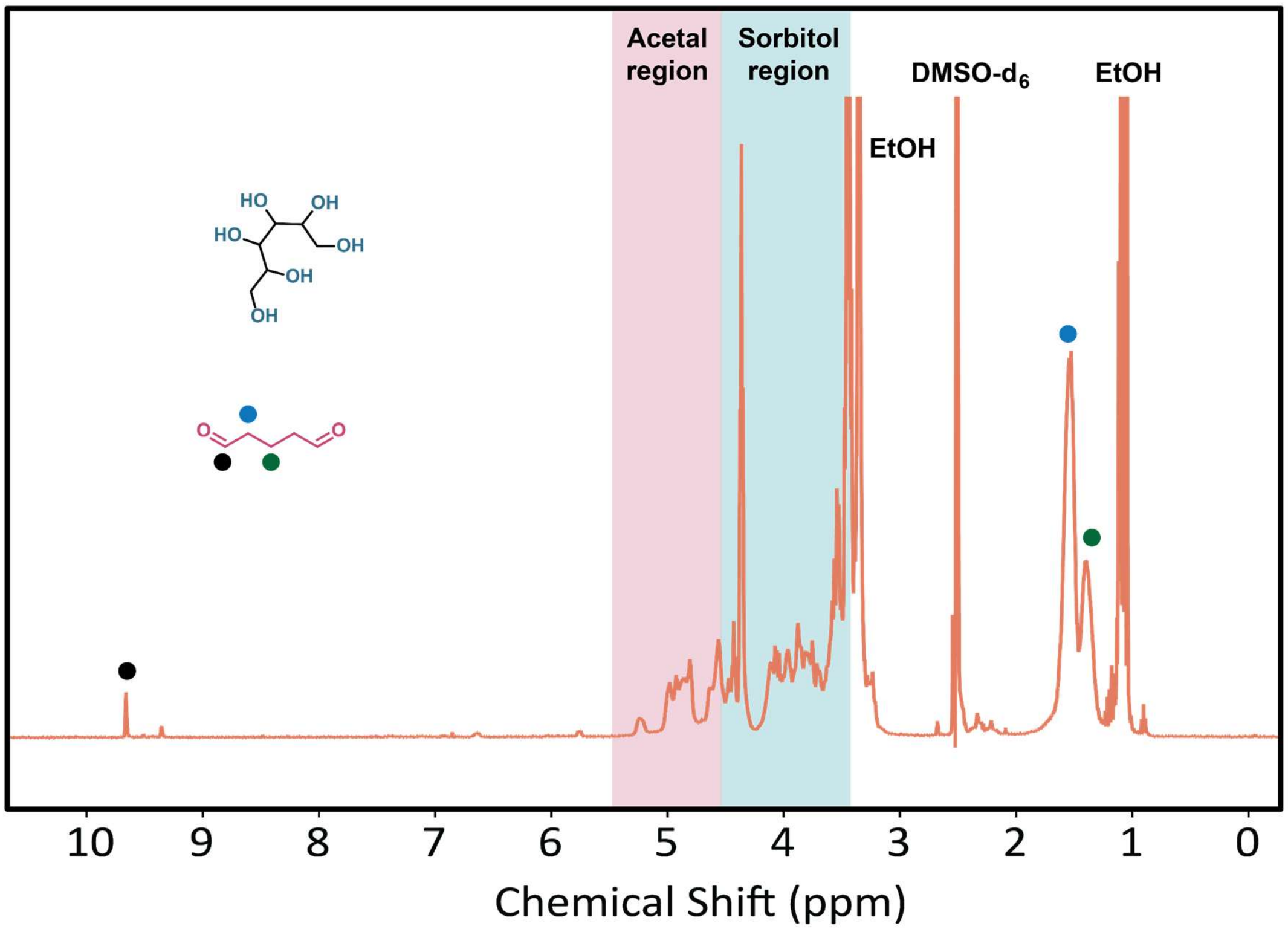

Figure S8: ^{1}H NMR spectrum of the depolymerization mixture obtained from AT treated with 2 M ethanolic H_3PO_4 for 15 h at 80 °C, with regions associated with acetal and sorbitol moieties highlighted in pink and blue (DMSO-d_6, 400 MHz).

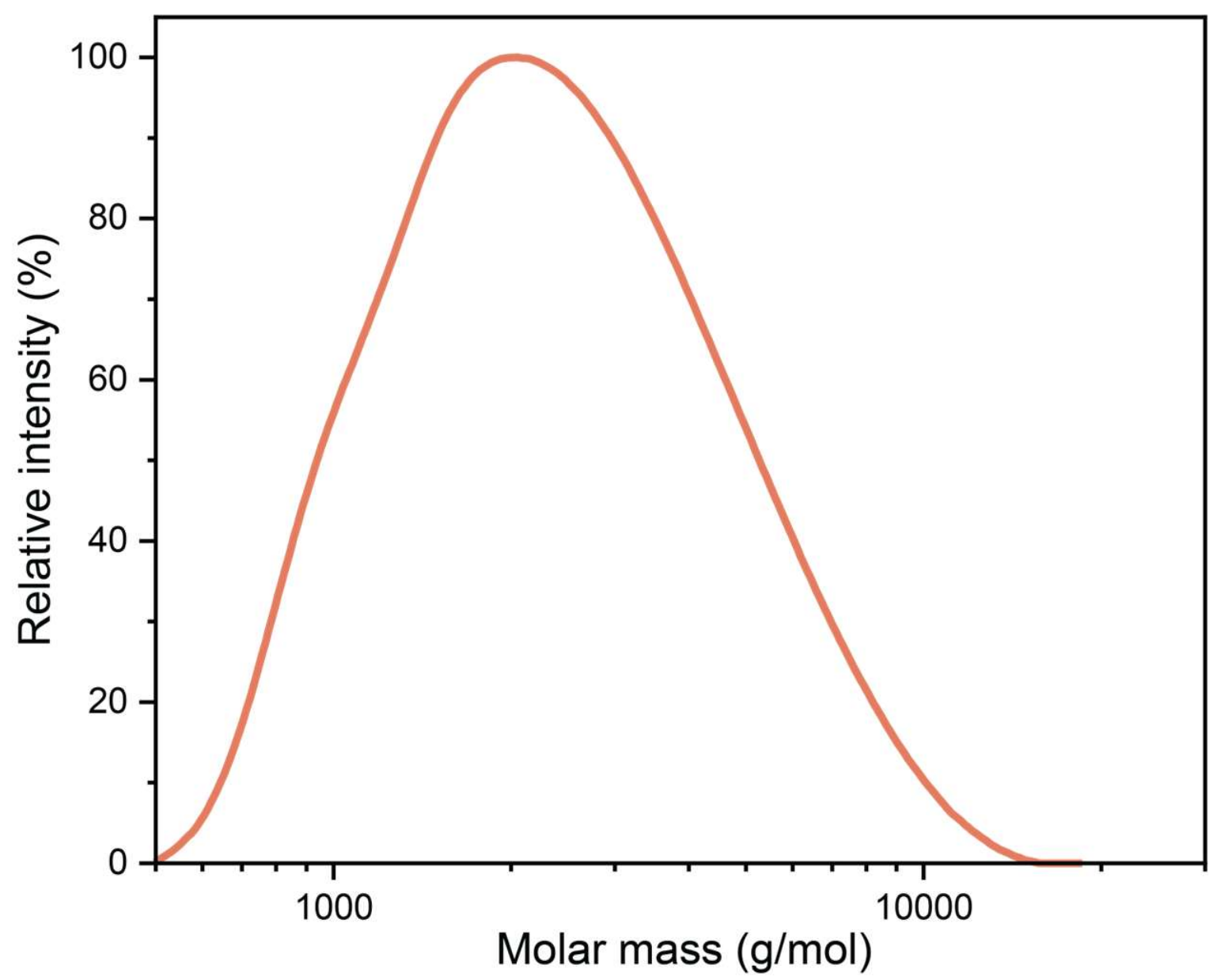

Figure S9. Gel permeation chromatography (GPC) trace of the depolymerization mixture measured in DMF using PMMA calibration standards.

Table S3: Properties of AT and AT-R obtained by DSC and tensile test

Sample	T_g (DSC) (°C)	Elongation at break (%)	Stress at break (MPa)	Young's modulus (GPa)
AT	96	4.9 ± 0.5	98.8 ± 6.6	3.3 ± 0.3
AT-R	102	3.8 ± 0.4	92.9 ± 14.6	3.3 ± 0.6

Synthesis and characterization of acetal thermoset composites

Table S4: Properties of AT GFAT and CFAT obtained by DMA and tensile test

Sample	T_g (DMA) (°C)	Elongation (%)	Stress(MPa)	Young's Modulus (MPa)
AT	114	4.9 ± 0.5	98.8 ± 6.6	3.3 ± 0.3
GFAT	94	4.0 ± 0.4	390 ± 24	13.1 ± 1.0
CFAT	98	2.7 ±.0.4	510 ± 60	22.6 ± 0.9

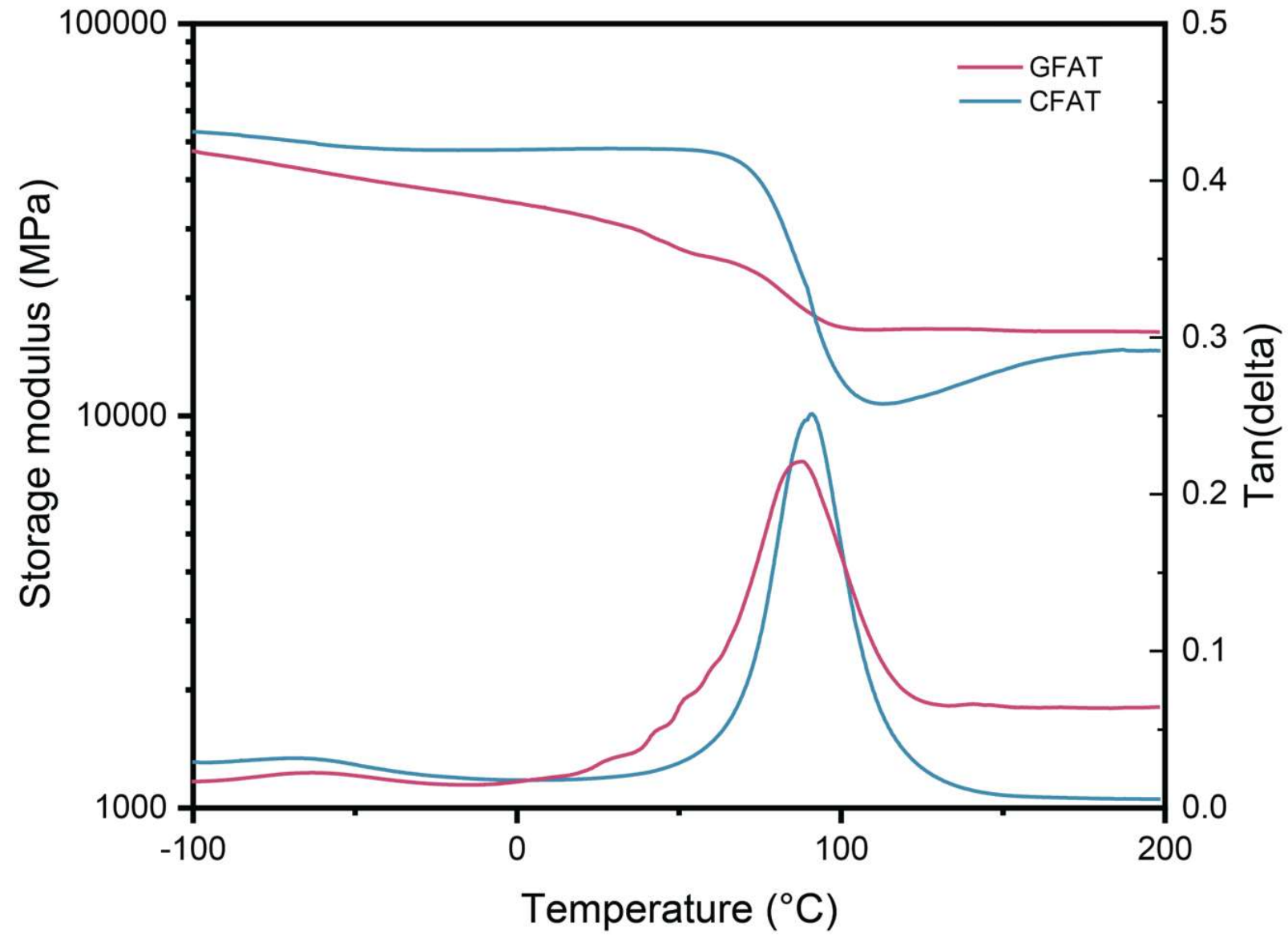

Figure S10. DMA bending analysis of GFAT and CFAT composites samples measured in duel-cantilever mode.

Acetal thermoset composites recycling and fiber recovery

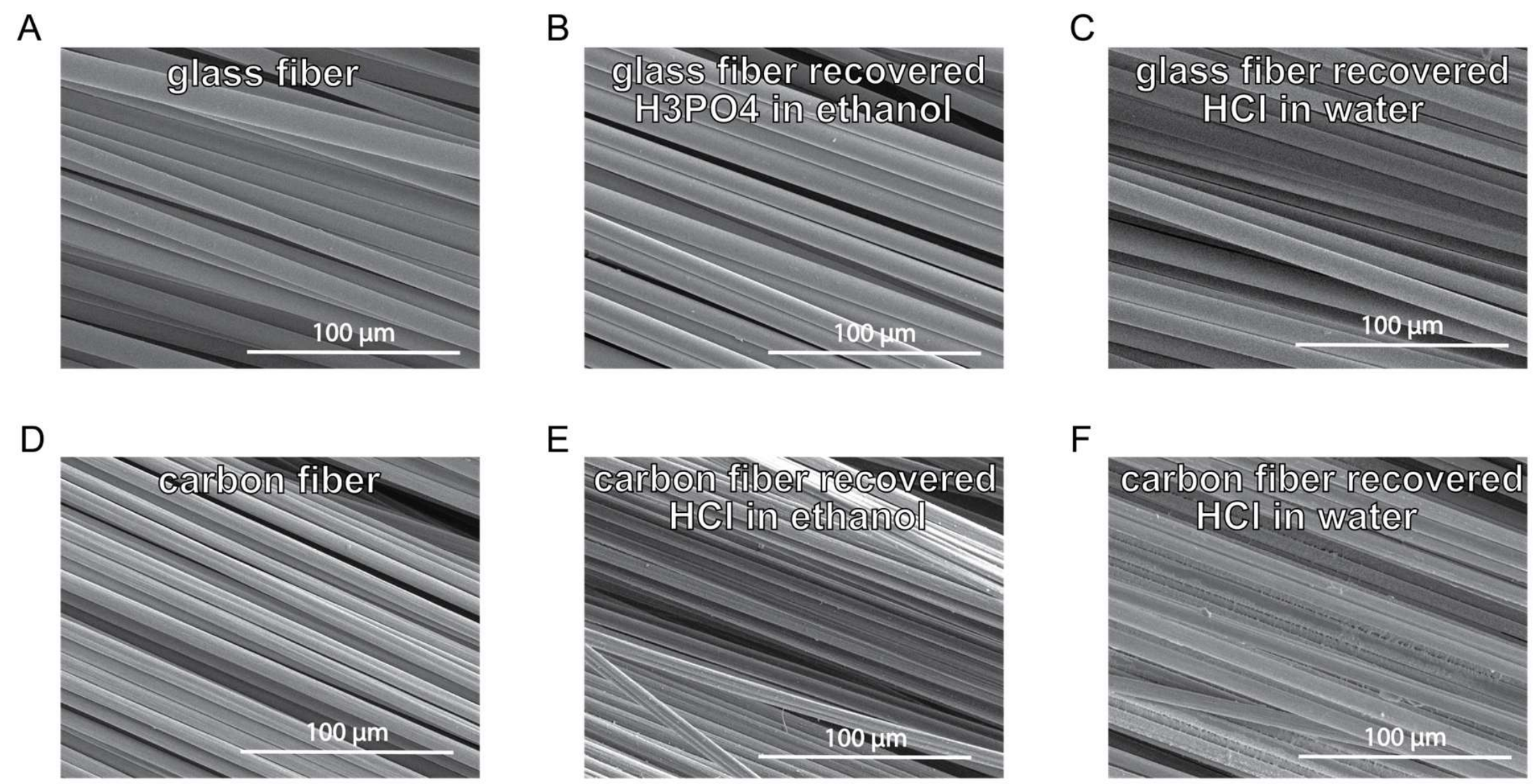

Figure S11. SEM images of virgin and recovered fibers from GFAT and CFAT composites: (A,D) virgin glass and carbon fibers, (B,C,E) corresponding fibers recovered after depolymerization of the acetal matrix, showing complete removal of resin and preservation of fiber morphology. (F) Carbon fiber recovered after HCl treatment in water, displaying significant residual matrix on the fibers.

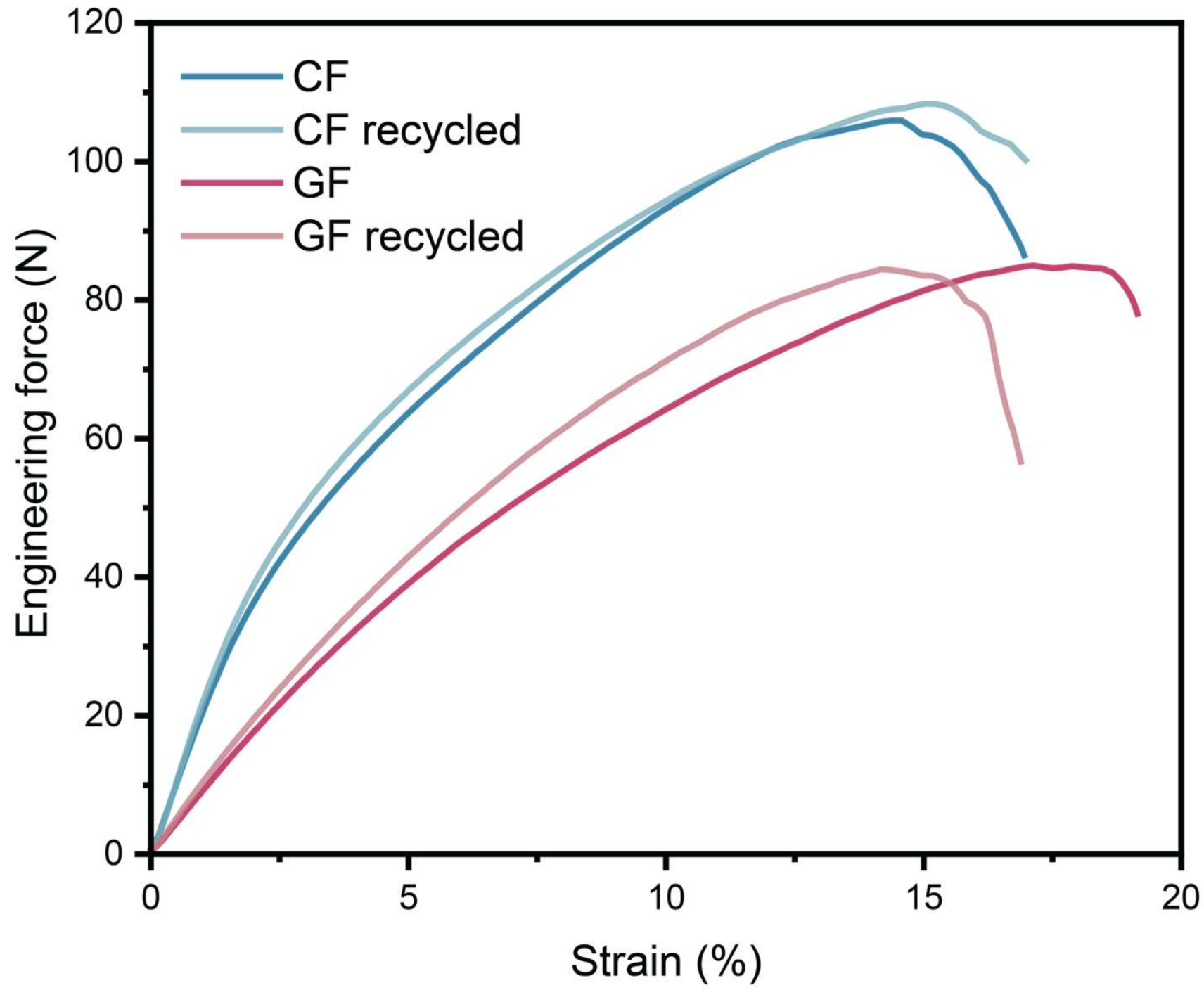

Figure S12. Representative force–strain curves of recovered glass and carbon fiber bundles compared with their virgin counterparts, demonstrating full retention of mechanical performance.

Table S5: maximum force and elongation at maximum stress of the recovered fibers

	Carbon fibers	Carbon fibers recovered	Glass fibers	Glass fibers recovered
Maximum force (N)	110 ± 8	111 ± 3	92 ± 7	92 ± 12
Elongation (%)	15.3 ± 0.6	14.1 ± 1.5	17.8 ± 1.7	15.4 ± 1.1
